# Deciphering the Code for Retroviral Integration Target Site Selection

**DOI:** 10.1371/journal.pcbi.1001008

**Published:** 2010-11-24

**Authors:** Federico Andrea Santoni, Oliver Hartley, Jeremy Luban

**Affiliations:** 1 Department of Microbiology and Molecular Medicine, University of Geneva, Geneva, Switzerland; 2 Swiss Institute of Bioinformatics, University of Geneva, Geneva, Switzerland; 3 Center for Advanced Studies, Research, and Development in Sardinia, Pula, Italy; 4 Department of Structural Biology and Bioinformatics, University of Geneva, Geneva, Switzerland; University of California San Diego, United States of America

## Abstract

Upon cell invasion, retroviruses generate a DNA copy of their RNA genome and integrate retroviral cDNA within host chromosomal DNA. Integration occurs throughout the host cell genome, but target site selection is not random. Each subgroup of retrovirus is distinguished from the others by attraction to particular features on chromosomes. Despite extensive efforts to identify host factors that interact with retrovirion components or chromosome features predictive of integration, little is known about how integration sites are selected. We attempted to identify markers predictive of retroviral integration by exploiting Precision-Recall methods for extracting information from highly skewed datasets to derive robust and discriminating measures of association. ChIPSeq datasets for more than 60 factors were compared with 14 retroviral integration datasets. When compared with MLV, PERV or XMRV integration sites, strong association was observed with STAT1, acetylation of H3 and H4 at several positions, and methylation of H2AZ, H3K4, and K9. By combining peaks from ChIPSeq datasets, a supermarker was identified that localized within 2 kB of 75% of MLV proviruses and detected differences in integration preferences among different cell types. The supermarker predicted the likelihood of integration within specific chromosomal regions in a cell-type specific manner, yielding probabilities for integration into proto-oncogene *LMO2* identical to experimentally determined values. The supermarker thus identifies chromosomal features highly favored for retroviral integration, provides clues to the mechanism by which retrovirus integration sites are selected, and offers a tool for predicting cell-type specific proto-oncogene activation by retroviruses.

## Author Summary

When HIV-1, murine leukemia virus (MLV), or other retroviruses infect a cell, the virus generates a DNA copy of the viral RNA genome and ligates the cDNA within host chromosomal DNA. This integration reaction occurs at sites throughout the host cell genome, but little is known about how integration sites are selected. We attempted to identify markers predictive of retroviral integration by comparing the genome-wide binding sites for more than 60 factors with 14 retroviral integration datasets. We borrowed Precision-Recall methods from the Information Retrieval field for extracting information from highly skewed datasets such as these. For MLV and other gammaretroviruses, strong association was observed with STAT1, acetylation of H3 and H4 at several positions, and methylation of H2AZ, H3K4, and K9. We generated a supermarker by combining high scoring markers. The supermarker localized within 2 kB of 75% of MLV proviruses and predicted the likelihood of integration within specific chromosomal regions in a cell-type specific manner. This study identified chromosomal features highly favored for retroviral integration. It also provides clues to the mechanism by which retrovirus integration sites are selected, and offers a tool for predicting cell-type specific proto-oncogene activation by retroviruses.

## Introduction

Retroviruses and retrotransposons are of profound importance to eukaryotic biology, evolution, and medicine. These retroelements constitute at least 40% of the mass of mammalian genomes [1] and 75% of the maize genome [2]. When retroelements are transcribed they remodel eukaryotic genomes by generating a cDNA and integrating it into locations scattered throughout the host cell genome [3], [4]. By doing so, retroelements have the potential to influence local gene expression or to promote recombination and generate deletion mutations [5]–[7]. In some cases they act *in trans* to catalyze retrotransposition of cellular RNAs, generating pseudogenes or new exons within existing genes [8], [9]. Since retrotransposon enhancer elements influence local gene expression, and retrotransposon silencing can vary from cell to cell, it has been proposed that retrotransposons contribute to the phenotypic variation that distinguishes genetically identical individuals [10]. Additionally, it has been suggested that programmed release from retroelement silencing accompanies metazoan development and leads to hypermutation in complex somatic tissues like the brain [11], [12].

Among retroelements, retroviruses have received much attention, in part due to their association with human disease. Basic studies concerning retroviral replication have greatly advanced understanding of the biochemistry of retrotransposition [4], [13]. A tetramer of the viral integrase protein (IN) [14] cleaves the ends of the viral cDNA to produce recessed 3′OH and free CA dinucleotides at the terminus of each long terminal repeat (LTR) [15]. IN catalyzes nucleophilic attack of host chromosomal DNA by the two free 3′-OH viral DNA ends, resulting in covalent attachment of the retroviral DNA strands to the host DNA [16]–[18]. The remaining free ends of the viral DNA are then repaired by host enzymes [19]–[21].

Study of HIV-1, the retrovirus that causes AIDS, has led to the development of drugs that block retrotransposition and alter progression to AIDS [22], [23]. Attempts to develop better therapies for HIV-1 would benefit from a deeper understanding of the integration mechanism. Gene therapy vectors based on another retrovirus, MLV, dramatically rescued children from a life-threatening illness, but a large percentage of the patients suffered from insertional activation of proto-oncogenes [24]–[28]. This lethal complication further emphasizes the need to better understand retroviral integration site selection in host chromosomal DNA.

Retroviruses establish proviruses at sites throughout the host cell genome, but integration is not random. Some regions are favored hundreds of times over others [29], [30]. For some retroviruses, transcribed regions are preferred [31], [32], though high-level, concurrent transcription at a given target gene inhibits integration [33]. Nucleosome-bearing DNA is targeted more efficiently than free DNA *in vitro* [34]–[37] perhaps because the integration machinery preferentially targets bent DNA [38]. Indeed, high-throughput sequencing experiments analyzing over 40,000 HIV-1 integration sites in cells show periodic distribution on predicted nucleosome positions, consistent with favored integration into outward-facing DNA major grooves in chromatin [39].

The retrotransposition mechanism, and integration site selection on a genomic scale, differs considerably from one class of retrovirus to another. HIV-1 infects non-dividing cells [40], [41] and integrates preferentially into transcriptionally active genes, all along the length of the gene [32], [42], [43]. In contrast, MLV integration requires mitosis [41], [44] and has a tendency to localize near promoters, 20% of the time within 2 kB of transcriptional start sites [31], [42]. Retroviral capsid (CA) is sufficient to determine whether a given virus infects non-dividing cells [45], [46] but both CA and IN contribute to integration site selection: an HIV-1 vector in which IN-coding sequences and a fragment of *gag* encompassing CA were replaced by the homologous MLV sequences exhibits the retrotransposition behavior of MLV [43].

Of the many host factors reported to interact with retroviral CA or IN [47]–[52], the lentiviral IN-interacting protein PSIP1/LEDGF/p75 [53]–[55] is the most informative regarding integration site selection. LEDGF promotes the infectivity of HIV-1 and related lentiviruses and influences integration site selection [56]–[59] perhaps by acting as a physical tether directing integration to the chromosomal sites this protein naturally occupies. In support of this model, fusion of heterogeneous chromatin binding domains to the part of LEDGF that binds IN redirected the site of HIV-1 integration [60]–[62]. The mechanism by which gammaretroviruses such as MLV preferentially target promoter regions is unknown.

We attempted to identify chromatin features predictive of retroviral integration site selection by exploiting ChIPSeq datasets. Compared to previous methods, this technology has brought profiles of human DNA binding factors and histone epigenetic modifications closer to genome-wide saturation [63]–[68]. Over 60 ChIPSeq datasets were compared with 14 retroviral integration data sets in order to develop tools for predicting viral integration sites throughout the genome with maximal predictive power.

## Results

### Development of methods for detection and display of associations between retroviral integration sites and chromatin features

To identify markers predictive of retroviral integration site selection, stringent associations were sought between ChIPSeq profiles for more than 60 chromatin-associated factors (Table 1) [63]–[69] and 14 retroviral integration site datasets (Table 2) [31], [43], [70]–[77]. Following a common convention in the retrovirus integration literature [78], association with a given marker was defined as integration within 2 kB (wi2kB) of the nearest marker on the linear sequence of the chromosome.

**Table 1:** ChIPSeq datasets from human cells used in this paper.

Cell type	ChIP Target	Reference
HeLa	STAT1	[63]
	h3k4m1	[63]
	h3k4m3	[63]
CD4^+^ T	aHistone methylations	[64]
CD4^+^ T	bHistone acetylations	[69]
HeLa	POLR2	[66]
HeLa	CTCF	[67]
CD4+ T	CBP	[65]
	MOF	[65]
	P300	[65]
	TIP60	[65]
	PCAF	[65]
	HDAC1	[65]
	HDAC2	[65]
	HDAC3	[65]
	HDAC6	[65]
HeLa	h3k9ac	[65]
	h3k16ac	[65]

a 25 different ChIPSeq profiles have been reported in this paper.

b 18 different ChIPSeq profiles have been reported in this paper.

**Table 2:** Retrovirus integration datasets in human target cells used in this paper.

Retrovirus	Target cell	Reference
MLV	HeLa	[31]
MLV	HeLa	[43]
MLV	CD4+ T	[71]
MLV	CD34+ hemato.	[74]
HIVmINmGAG	HeLa	[43]
HIVmIN	HeLa	[43]
HIVmGAG	HeLa	[43]
HIV	HeLa	[43]
HIV	CD4+ T	[75]
PERV	HEK293	[77]
XMRV	DU145	[76]
HTLV	HeLa	[73]
ASLV	HeLa	[70]
FV	CD34+ hemato.	[72]

The proviruses in the datasets used here (Table 2) were cloned from host genomic DNA using restriction enzymes, each of which has the potential to introduce a bias [79]. Therefore, as described in the literature [42], [43], [78], [80], each integration site was matched to ten control sites designed to exhibit the same bias as the experimental set: control sites were placed the equivalent distance from randomly chosen recognition sites of the restriction enzyme that was used to clone the provirus (see Methods). No distortion of the results by the control datasets was evident, in that identical values for provirus association with a given chromatin feature were obtained using 10 different randomly-generated control datasets.

Integration datasets are generally compared with control datasets using Fisher's exact test and reported as the p-value [42], [43], [77], [80]. Since significance determination is dependent upon dataset size, these measures can be easily conflated, generating extraordinarily low p-values and making it difficult to compare the importance of two factors [78]. Receiver operating characteristic area methods (ROC) have also been used to identify associations [78], [80], [81], but these methods also have drawbacks when it comes to discriminating between markers for retroviral integration. With the datasets used in these studies, the number of true negatives (control sites not associated with the marker) is considerably higher than the number of false positives (control sites associated with the marker). Given that the false positive rate = false positives / [false positives+true negatives], two markers which differ by as much as 10-fold in terms of the number of false positives will fail to be differentiated from one another using ROC [82].

To address the problems associated with the analysis of these highly skewed data sets, we borrowed the concepts of Precision and Recall from the field of Information Retrieval [82]–[84]. In the context of this discussion, Precision is defined as the number of experimentally-determined integration sites associated with a marker divided by the sum of all associated experimental and all associated control sites (see Methods). Recall is the number of marker-associated experimental integration sites divided by all experimental integration sites. The F_β_ score, a convenient way to aggregate Precision and Recall, is the weighted harmonic mean of the two measures [85]. Usual values for β are 0.5, 1 or 2 [86]. To limit the influence of true negatives in the analysis of these skewed datasets, we emphasized Precision over Recall by setting β = 0.5. The F score tracks better with statistical significance when β = 0.5, than 1 or 2 (see the comparison of results using different values for β, as well as with other metrics, described below, as well as Text S1). Moreover we normalized the number of false positives with respect to the number of experimental integration sites so as to make the F score independent of control sample size. For the analysis here, markers with F scores between 0.5 and 1 were considered to be associated with integration sites.

**Figure 1:**
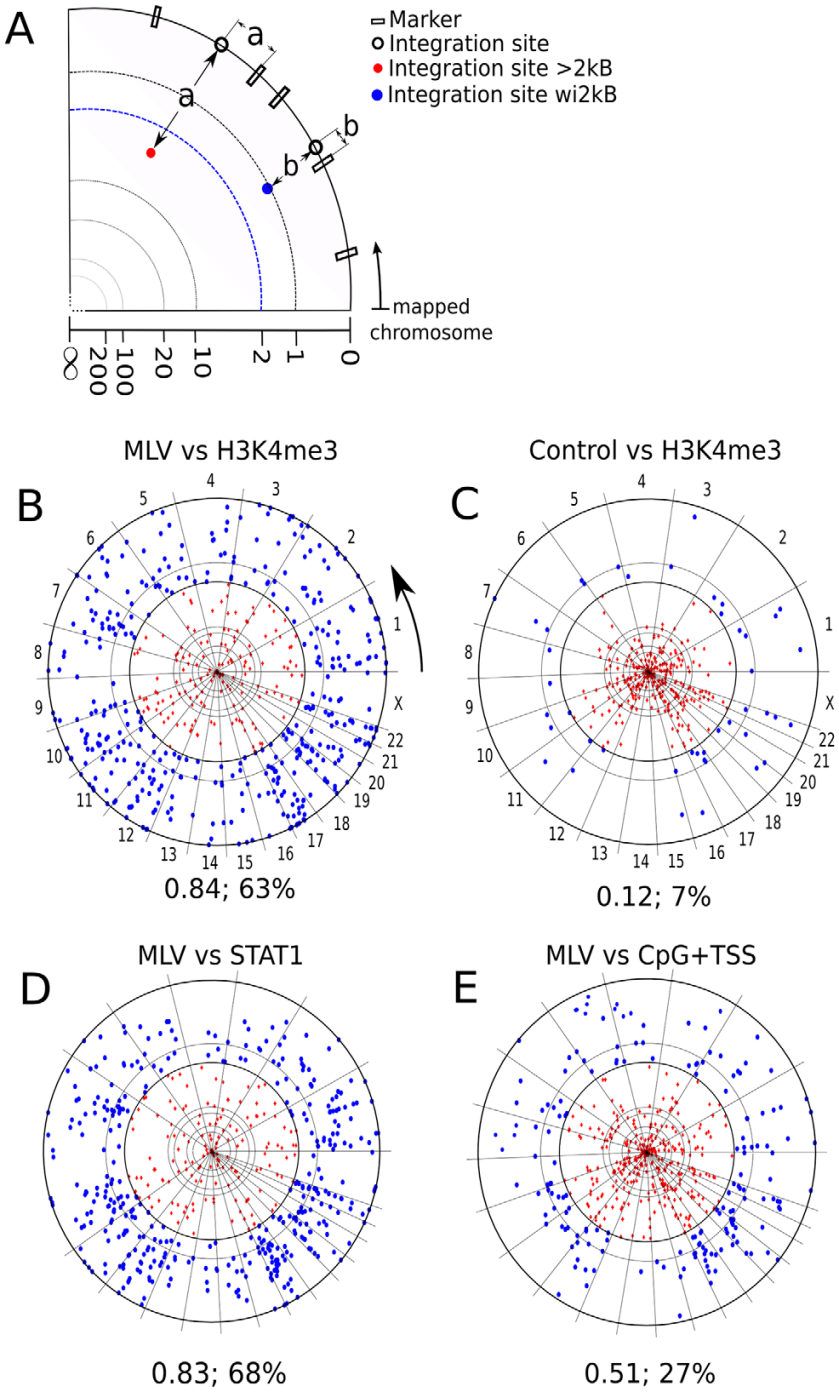
Visualization of association between retroviral integration sites and chromosomal markers. (**A**) Construction of chromosome projection mandalas to visualize the proximity of individual proviruses to the nearest marker on the chromosome. The linear sequence of each human chromosome was linked and circularized. Proviral integration sites were located on the circle according to their position on each chromosome (empty circles) and then a marker (filled circles) was placed towards the center of the circle, at a distance from the perimeter that was equal, in log scale from 0 to 1 megabase, to the distance from the closest marker (empty boxes). Blue filled circles represent proviruses that were within 2kB from the nearest marker; red circles represent proviruses that are >2kB from the nearest marker. Examples of chromosome projection mandala for (**B**) MLV (Lewinski et al. 2006) versus H3K4me3, the arrow indicates the chromosomal mapping direction (**C**) Control versus H3K4me3 (**D**) MLV versus STAT1 and (**E**) MLV versus CpG+TSS. The number of MLV proviruses analyzed in this dataset (Lewinski et al. 2006) was 588. The F score and the percentage of proviruses within 2 kB are presented under each mandala.

To visualize genome-wide association of proviruses with potential markers, chromosome projection mandalas were developed (Figure 1A, see Methods). Each dot on the mandala represents a retroviral integration site with the following polar coordinates: angular distance corresponds to genomic location on the indicated chromosome; radial distance from the contour of the circle is the distance in nucleotides from the nearest site of the marker in question, log-scaled from 0 to 1 megabase.

### Association of retroviral integration sites with ChIPSeq datasets

**Figure 2:**
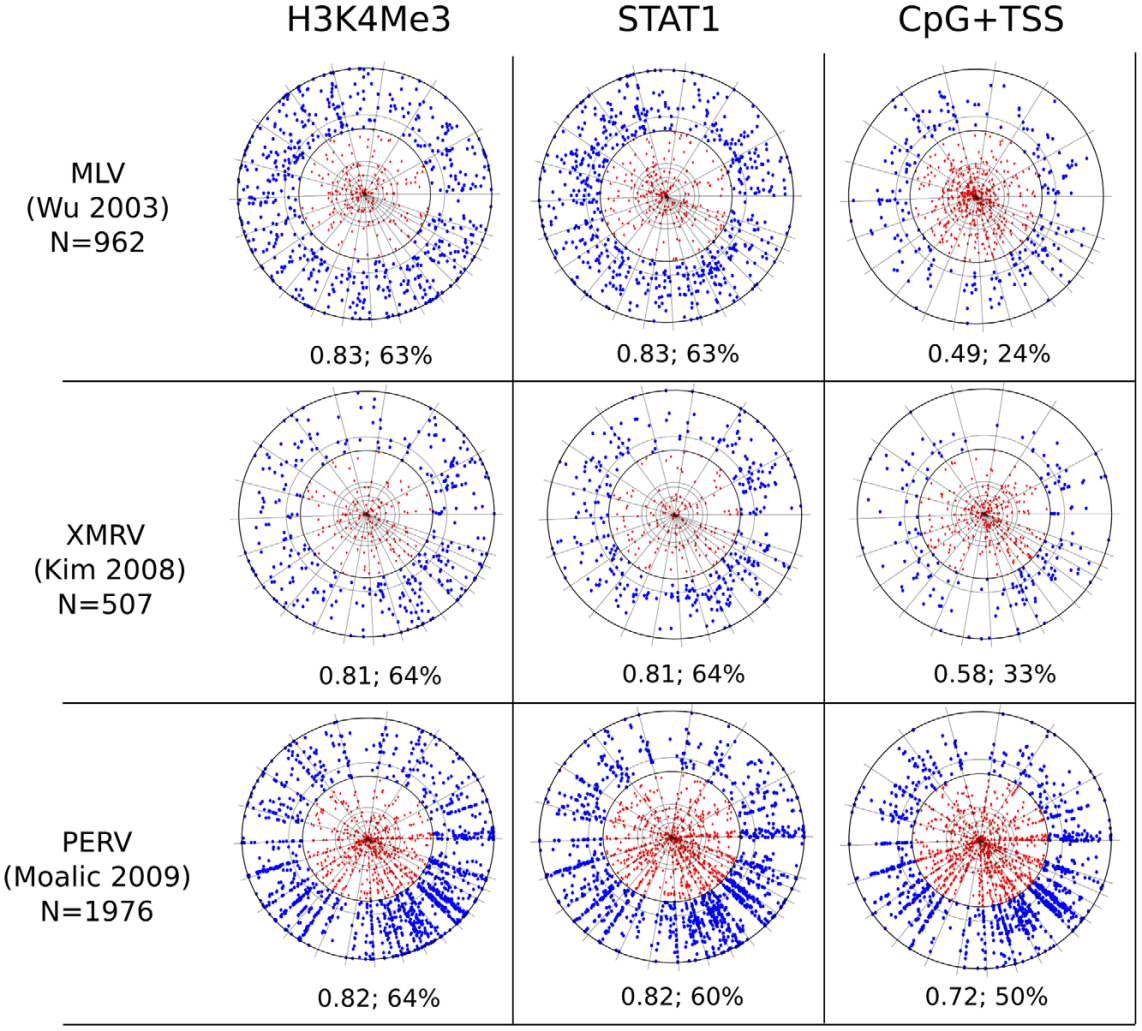
Chromosome projection mandala and F score calculated within 2 kB for the indicated markers (columns) versus the indicated proviruses (rows). The source of the provirus datasets is listed (see Table 2 and the text) and N indicates the number of proviruses considered for each analysis. MLV [31] proviruses were cloned from HeLa cells, XMRV proviruses from DU145, and PERV proviruses from HEK 293. H3K4me3 and STAT1 ChIPSeq datasets were from HeLa (see Table 1 and text). The F score and the percentage of proviruses within 2 kB are presented under each mandala.

**Figure 3:**
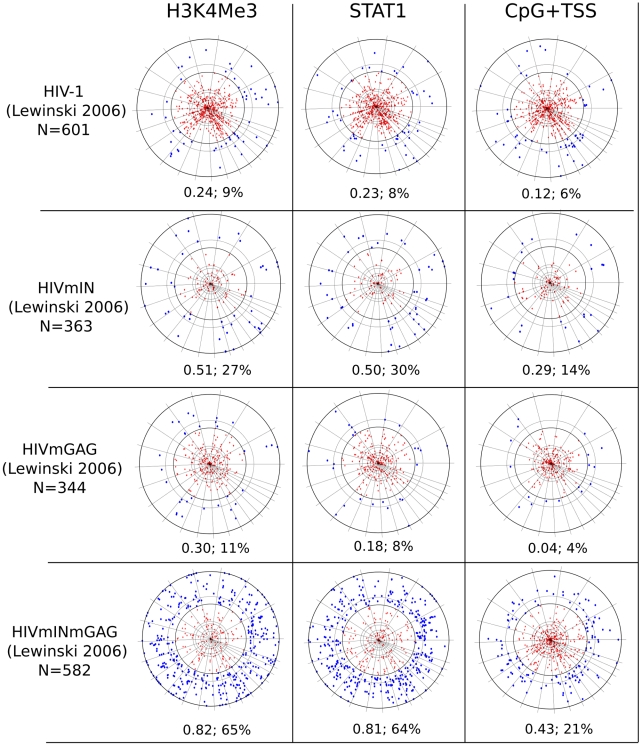
Chromosome projection mandala and F score calculated within 2 kB for the indicated markers (columns) versus the indicated proviruses (rows). All proviruses were cloned from HeLa cells (Table 2 and text). H3K4me3 and STAT1 ChIPSeq datasets were from HeLa cells (Table 1). N indicates the number of specific proviral integrations considered for each analysis. The F score and the percentage of proviruses within 2 kB are presented under each mandala.

Currently, the best chromosomal marker for retroviral integration site selection is the association of CpG islands and transcription start sites (CpG+TSS) with gammaretroviruses [31], [43], [71]. By examining published datasets for MLV, 21 to 27% of integration sites fall within 2 kB (wi2kB) of CpG+TSS, with probabilities <3×10^−22^ to <4×10^−42^ (Table 3). Despite these extremely low p-values, F scores calculated for these datasets fall between 0.36 to 0.51 (Table 3 and Figure 1E), indicating that CpG+TSS is not a powerful predictor of MLV integration sites. Stronger association with CpG+TSS was observed with porcine endogenous retrovirus, PERV (50% wi2kB; p<10^−250^; F score 0.72), and xenotropic MuLV-related virus, XMRV (33% wi2kB; p<10^−46^; F score 0.58), two viruses from the same gammaretrovirus family as MLV (Table 3 and Figure 2). No significant association with CpG/TSS was observed for proviruses generated by non-gammaretroviruses, including HIV-1, for which the F score was 0.11 (Table 3, Figure 3), or with ASLV, HTLV, or Foamy virus (Table 3, Figure S1).

**Table 3:** Association of retroviral integration sites with some ChIPSeq profiles.

Provirus Dataset	CpG+TSS	H3K4 me3	H3K4 me1	STAT1	POL II	CTCF
MLV HeLa [31]	24%; 0.49; 6E-24	63%; 0.84; <1E-350	88%; 0.80; 1E-240	63%; 0.83; 1E-310	46%; 0.70; 1E-198	5%; 0.26; N.S.
MLV HeLa [43]	27%; 0.51; 4E-42	68%; 0.83; 1E-249	90%; 0.78; 1E-226	68%; 0.83; 4E-324	49%; 0.71; 2E-164	7%; 0.19; 4E-5
MLV CD4+T [71]	21%; 0.36; 3E-22	65%; 0.82^a^; 1E-110	75%; 0.80^a^; 1E-90	47%; 0.73; 2E-46	34%; 0.64; 2E-43	3%; 0.17; N.S.
HIV [43]	6%; 0.11; N.S.	9%; 0.24; N.S.	48%; 0.60; 1E-31	8%; 0.27; N.S.	6%; 0.16; N.S.	2%; 0.06; N.S.
HIV mIN [43]	14%; 0.29; N.S.	27%; 0.50; 1E-14	49%; 0.51; 1E-11	30%; 0.51; 1E-12	13%; 0.36; 2E-6	5%; 0.17; N.S.
HIV mGAG [43]	4%; 0.04; N.S.	11%; 0.30; N.S.	43%; 0.56; 1E-11	8%; 0.11; N.S.	3%; 0.14; N.S.	1%; 0.04; N.S.
HIV mINmGAG [43]	21%; 0.43; 4E-14	65%; 0.82; 1E-221	89%; 0.79; 1E-150	64%; 0.81; 1E-183	33%; 0.67; 4E-101	4%; 0.16; N.S.
PERV [77]	50%; 0.72; <1E-350	64%; 0.82; <1E-350	79%; 0.78; <1E-350	60%; 0.82; <1E-350	56%; 0.70; <1E-350	12%; 0.3; 3E-40
XMRV [76]	33%; 0.58; 1E-46	64%; 0.81; 8E-175	83%; 0.76; 1E-144	64%; 0.81; 9E-171	53%; 0.75; 1E-135	7%; 0.36; 2E-3
HTLV [73]	8%; 0.21; N.S.	30%; 0.59; 1E-15	62%; 0.70; 6E-26	31%; 0.60; 4E-15	13%; 0.39; 1E-6	6%; 0.22; N.S
ASLV [70]	10%; 0.10; N.S.	16%; 0.43; 1E-4	39%; 0.56; 1E-4	13%; 0.37; N.S.	6%; 0.13; N.S.	2%; 0.08; N.S.
FV [72]	11%; 0.27; 2E-5	17%; 0.42; 1E-17	39%; 0.56; 1E-22	17%; 0.44; 6E-17	9%; 0.28; 1E-14	4%; 0.17; N.S.

Values indicate percent of integration sites within 2 kB of the indicated factor; the F_0.5_ score; and the significance (p-value). N.S. means p value>0.01.

a ChIPSeq profiles from CD4+ T cells. All other ChIPSeq profiles from HeLa cells.

**Figure 4:**
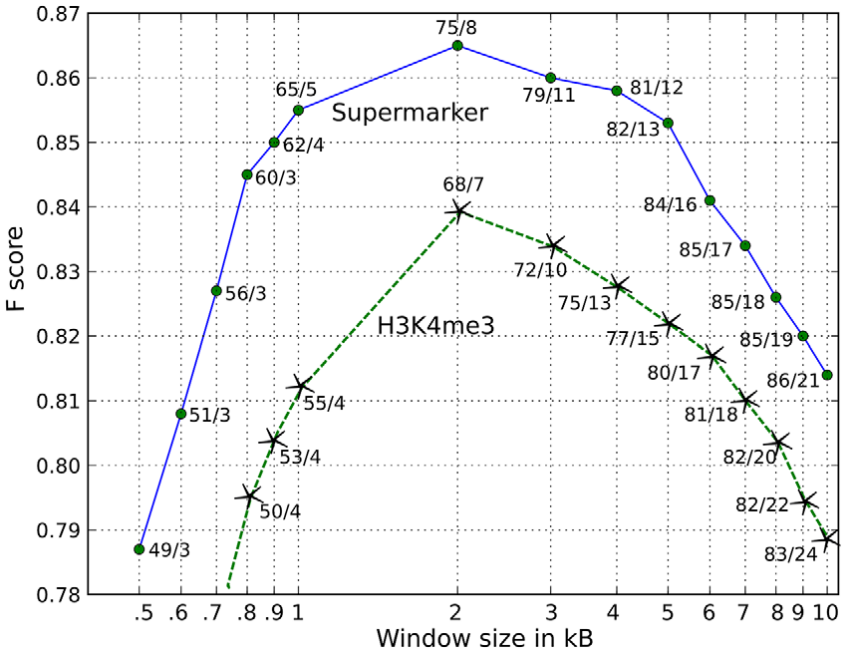
Influence of window size on the F score. Association (F score) between MLV proviruses [43] and either H3K4me3 (green dashed line with stars) or the supermarker in HeLa cells (solid blue line with solid circles) as a function of window size in kilobases. The true positive fraction versus the false positive fraction is shown for each point.

ChIPSeq datasets for 60 chromatin-associated factors (Table 1) were compared with 14 provirus datasets for MLV, PERV, XMRV, HIV-1, HTLV-1, ASLV, Foamy virus, and HIV/MLV chimeras (Table 2). Acetylation of H3 and H4 at several positions, and methylation of H2AZ, H3K4, and K9, were strongly associated with gammaretroviral integration sites, all with F scores >0.80 (Figures 1 and 2, Table 3 and Tables S1 and S2). H3K4me3 in particular was strongly associated with MLV integration sites (68% wi2kB; p<10^−324^; F score 0.83) and with the integration sites of PERV (60% wi2kB; p<10^−350^; F score 0.82) and XMRV (64% wi2kB; p<10^−170^; F score 0.81) (Figures 1 and 2, Table 3). The effect of window size on the F score was examined for factors strongly associated with MLV and the other gammaretroviruses. Interestingly, the F score was maximal when it was calculated using a window of +/−2 kB for proviruses flanking the sites of these chromatin features (Figure 4).

In contrast to the gammaretroviruses, HIV-1 integration sites were not associated with H3K4me3 (9% wi2kB; p>0.05; F score 0.21)(Figure 3 and Table 3). Among the markers for which ChIPSeq datasets were available from HeLa cells, H3K4me1 had the strongest association with HIV-1 proviruses (48% wi2kB; p<10^−31^; F score 0.6), though H3K4me1 was the sole chromatin marker that yielded F score values greater than 0.5 across all queried viruses (Table 3, Table S3). H3K4me3, and other chromatin modifications linked to transcriptionally active promoters [64], [87]–[89], were reported to be associated with HIV proviruses when a window of 50 kB flanking the proviruses was considered [81], [90]. This could be explained by the fact that HIV-1 proviruses localize to active transcription units with equal distribution along the length of the genes [32], [42], [43], and that the size of the average transcription unit is on the order of tens of kilobases.

**Figure 5:**
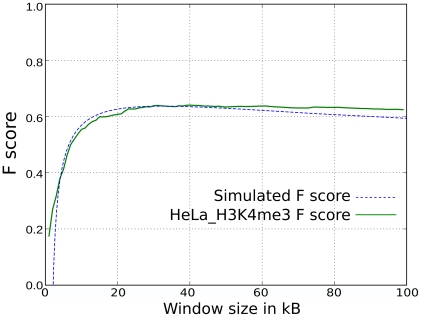
Association (F score) between HIV-1 proviruses and two markers as a function of window size in kB. The first marker is H3K4me3 sites in HeLa (green solid line). The second is a virtual marker placed in the promoter region of transcribed genes in HeLa cells (blue dashed line), assuming a uniform distribution of integration sites in transcribed regions. The median length of transcribed genes in HeLa is ∼20kB.

To examine this further, the F score for HIV-1 versus H3K4me3 in HeLa cells was plotted as a function of window size (Figure 5). For comparison, a similar plot was generated for a hypothetical marker at the TSS of transcribed genes in HeLa cells, taking into account the length of these genes, and considering a uniform distribution of proviruses on each gene. For both H3K4me3 and the hypothetical TSS marker, the F score plateaued at a window size of 20 kB, the median gene length. Thus if the window size is large enough to encompass the TSS and half of the gene length, the F score becomes significant. This could explain the window-size dependence of HIV-1 association with H3K4me3.

We also analyzed an integration site map for an HIV-1 vector in which IN-encoding *pol* sequences and part of *gag* were replaced by homologous sequences from MLV [45]. It was shown previously that substitution of these two viral components from MLV is sufficient to change the integration site preference of HIV-1, such that it targets TSS with a frequency like MLV [43]. Replacement with these MLV genes was sufficient for HIV-1 proviruses to associate with methylated histones (65% wi2kB, p<10^−182^, F score 0.82) in a manner that was indistinguishable from MLV (Figure 3).

### STAT1 association with gammaretroviruses

A remarkable association was found between MLV integration sites and STAT1 binding sites in IFN-γ stimulated HeLa cells (68% wi2kB; p<10^−324^; F score 0.83) (Figure 1 and 2, Table 3). Strong association with STAT1 binding sites was also observed for porcine endogenous retrovirus (60% wi2kB; p<10^−350^; F score 0.82) and XMRV (64% wi2kB; p<10^−170^; F score 0.81). Interestingly, if MLV was compared with STAT1 bindings sites in HeLa cells that had not been treated with IFN-γ the association was greatly decreased (34% wi2kb; p<10^−120^, F score: 0.69). HIV-1 proviruses showed no association with STAT1 (8% wi2kB; p>0.4; F score 0.27). Substitution of HIV-1 IN and parts of *gag* with the corresponding genes from MLV was sufficient for HIV-1 proviruses to associate with STAT1 binding sites (64% wi2kB, p<10^−182^, F score 0.81) (Figure 3, Table 3).

Attempts to detect a protein-protein interaction between STAT1 and MLV IN were unsuccessful. STAT1-deficient cell lines, either *Stat1* ^−/−^ mouse embryonic fibroblasts [91], HeLa cells with stable STAT1 knockdown using lentiviral vectors [92], or well-characterized, STAT1 mutant, HT1080 cells [93], were challenged with MLV and, as a control, HIV-1. No clear defect associated with STAT1-deficiency was detected when MLV infectivity was compared with HIV-1 (data not shown). These results suggest that STAT1 itself is not directly responsible for MLV integration site preference but that its chromatin preferences resemble those of MLV.

### The F score is robust and highly discriminating

**Figure 6:**
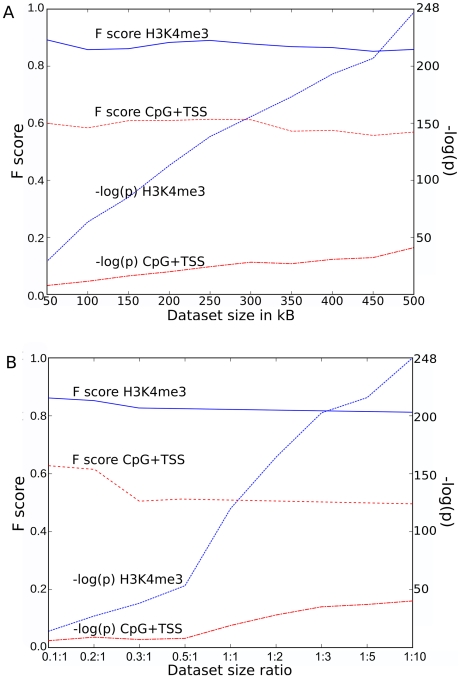
Stability of F score as function of dataset size. (**A**) Plot of the absolute value of the p-value exponent (right Y scale) or the F score (left Y scale) for H3K4me3 or CpG+TSS, as a function of MLV provirus dataset size. The experimental MLV dataset size (x-axis) was reduced by random sampling and the ratio of control dataset points was fixed at 10. (**B**) Examination of the same parameters as a function of the ratio between experimental and control dataset size (x-axis). The experimental dataset size was reduced by random sampling from 1∶1 down to 0.1∶1. From 1∶1 up to 1∶10 the control dataset size was proportionally increased by matched random generation.

The stability of the F score for H3K4me3, an excellent marker, and for TSS/CpG, a poor marker, was examined as the size of a dataset containing 588 MLV proviruses [43] was decreased. The ratio of the size of the provirus dataset with respect to the control dataset was fixed at ten. While the p-value varied enormously as the size of the provirus dataset decreased, the F score was constant for both H3K4me3 and TSS/CpG over the full range from 50 to 500 proviruses (Figure 6A). The size of the provirus dataset was then fixed at 588 [43] and the F score was plotted versus the ratio (from 0.1 to 10) of the experimental and control datasets. Under these conditions the F score for either factor was constant except for a small increase when the ratio of the experimental to control datasets decreased below 0.3 (Figure 6B). The p-value for H3K4me3 changed markedly with the change in ratio of the datasets. Thus, while the p-value is strongly biased by the size of the provirus dataset or by the ratio of experimental to control sites, the F score is a remarkably stable measure. Similar stability was observed for the F score of all markers as compared to all proviral integration datasets (data not shown).

**Figure 7:**
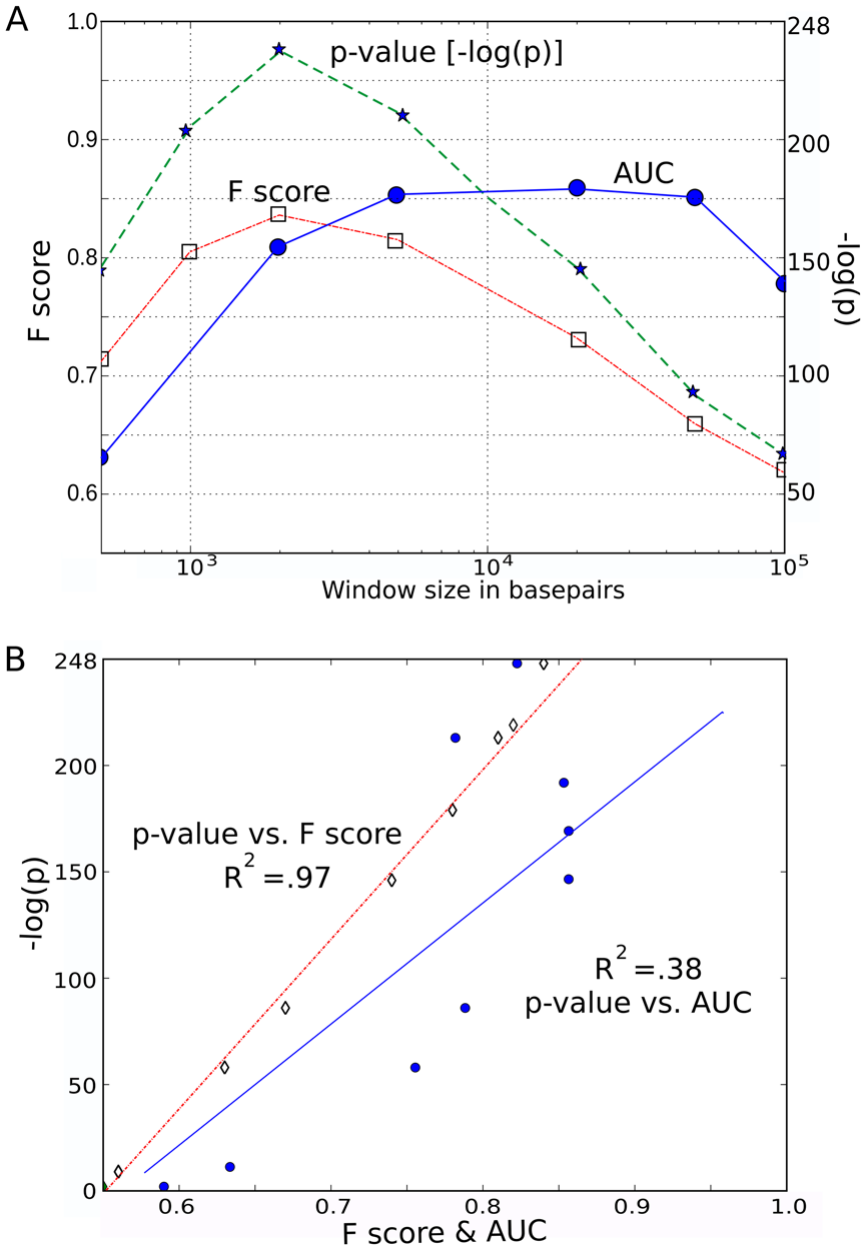
Effect of the window size on F score. (**A**) Plot of Area Under Curve (AUC) or F score (both on left Y scale) or the absolute value of the p-value exponent (right Y scale) for MLV with respect to H3K4me3 as a function of window size in basepairs. (**B**) Pearson correlation for AUC or F score (both on X axis) versus the absolute value of the p-value exponent (Y axis).

**Figure 8:**
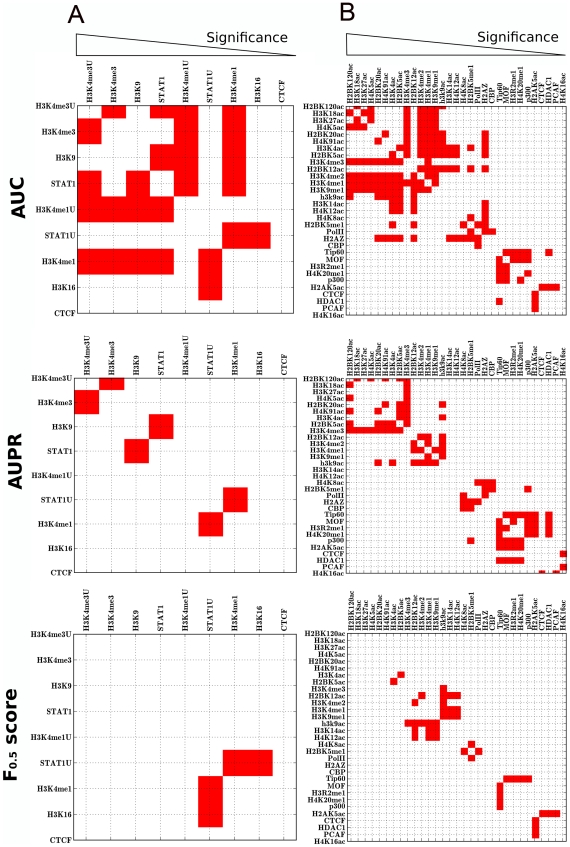
Comparison of different methods for ranking markers associated with integration. Markers for MLV integration in HeLa cells (**A**) or in CD4+ T cells (**B**) were ranked by Area Under Curve (AUC), Area Under Precision and Recall Curve (AUPR), or using the F_0.5_ score. The rankings obtained by these methods were compared with the ranking obtained by the Fisher's exact test: each crosslink between markers in the grid represents a comparison. Red squares indicate when the ranking calculated by the specified metric disagrees with the ranking calculated by significance. Markers were arranged in order of decreasing significance (from left to right).

As demonstrated for the F score (Figure 6), the area under the curve (AUC) ROC method used previously to evaluate markers associated with retroviral integration sites [78], [80], [81] is a robust measure that is insensitive to dataset size. Like the F score, AUC(ROC) also works well to assess markers that are weakly or moderately associated with integration sites (Text S1). But, as demonstrated for the highly associated marker H3K4me3, AUC(ROC) does not respond to the increase in false positives that is expected with increasing window size (Figure 7A). Moreover, this insensitivity to false positives leads AUC(ROC) to overestimate the association of markers that are more common in the genome. Consequently, AUC ranks markers differently from statistical significance, as shown in Figure 8 and discussed in more detail in Text S1. In contrast, the p-value and the F_0.5_ score incorporate an adjustment for the increase in false positives as window size increases, and both measures achieve a maximal value at a window size of 2 kB (Figure 7A). A standard regression plot shows that the F_0.5_ score tracks with the p-value almost perfectly (R^2^ = 0.97), whereas the AUC(ROC) diverges considerably (R^2^ = 0.37) (Figure 7B). The F_0.5_ score and the p-value adjust similarly for the increasing number of false positives.

Indeed among a set of measures that included F_0.5_, F_1_, F_2_, Area Under Curve (AUC), Area Under Precision/Recall (AUPR), Odds Ratio (OR), Shannon Mutual Information (SMI), and Difference of Proportions (DOP), the F_0.5_ score showed the strongest link with statistical significance (see Methods). We analyzed one of the MLV integration dataset in HeLa cells [43] (the same results were obtained using the other HeLa dataset [31]) and the MLV integration dataset in CD4+ T cells [71]. The strength of association of 9 significant markers (in terms of p-value) from HeLa cells, and 31 significant markers from CD4+ T cell, was assessed. Markers were ranked according to each of the above methods and the results of each were compared with the ranking obtained using significance −log(p value). This was done by fixing the matched control data set size at 10-times the experimental dataset size and using window sizes of 2, 5, 10, and 20 kilobases. Results for the analysis are reported in Table 4 and in Text S1.

**Table 4:** Comparison of different methods for ranking markers of MLV integration.

Provirus Dataset	Window Size	AUC	AUPR	F_0.5_	F_1_	F_2_	OR	SMI	DOP
HeLa [43]	2K	0.80	0.88	0.95	0.83	0.80	0.83	0.95	0.80
	5K	0.73	0.91	0.95	0.73	0.70	0.75	0.95	0.68
	10K	0.68	0.93	0.95	0.83	0.66	0.73	0.91	0.65
	20k	0.68	0.78	1.00	0.83	0.60	0.60	1.00	0.59
CD4+T [71]	2K	0.88	0.91	0.96	0.87	0.85	0.81	0.95	0.84
	5K	0.85	0.91	0.95	0.81	0.76	0.89	0.95	0.76
	10K	0.82	0.89	0.95	0.81	0.74	0.92	0.95	0.72
	20k	0.81	0.90	0.92	0.87	0.70	0.88	0.94	0.66

Similarity of the ranking of integration markers obtained by each metric with that yielded by Fisher's statistical significance. The formula used to calculate the similarity is in the methods. By this formula, 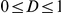, and *D* = 1 when the ranking perfectly matches that obtained by significance. AUC - Area Under the Curve, AUPR - Area Under Precision and Recall curve, F - F score at β = 0.5, 1, 2, OR - Odd Ratio, SMI - Shannon Mutual Information, DOP - Differences Of Proportions.

Several conclusions can be drawn from this analysis. Concerning markers that were highly associated with proviruses, the ranking yielded by the F_0.5_ score closely tracked with significance (Table 4). By increasing the weight of recall over precision by increasing the beta value (F_1_ or F_2_) the F score tracked less well with significance (it was the F_0.5_ score that was used throughout this manuscript). The SMI also tracked well, but, unlike the F score, the results with this method vary with dataset size (see Text S1). The AUC, OR, AUPR, and DOP were clearly not as good as the F_0.5_ score.

Concerning markers that are moderately or weakly associated with proviruses (Text S1), the ranking based on the F_0.5_ score was similar to that obtained by significance, AUC, AUPR, OR, or DOP (Table 4). SMI scored less well for these markers.

Figure 8 visualizes the deviation of AUC, AUPR or F_0.5_ from significance. Red squares indicate cases in which the ranking calculated by the specified metric differs from the rank obtained by significance. All results indicate that, for the datasets evaluated here, the F_0.5_ score is a superior measure at discriminating among factors for differences in magnitude of association with genomic sites of integration.

### Generation of a supermarker for retrovirus integration

Given the effectiveness of the F score for identifying and ranking individual factors associated with retrovirus integration site selection, markers with the best F scores were combined in an attempt to generate a supermarker (see Methods for more details). An estimate of the probability of proviral integration into the host genome (P(V)) was derived based on the genomic distribution of combinations of ChIPSeq peaks for the best scoring markers with respect to particular experimental provirus datasets. The resulting probability mass function (at base- pair resolution) is
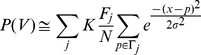
(A)where V is the set of proviral integration sites, F_j_ is the F score associated with each marker M_j_, for the set of peaks Γ_j_. x is the physical position on chromosomal DNA and K is a normalization constant. From this composite distribution, the peaks with the largest amplitude were identified, and the subset of peaks yielding the maximal F score in the test dataset was defined as the supermarker peak set.

Two strategies were used to validate the supermarker procedure. First we calculated the supermarker and the relative peak set on each single proviral dataset and then we evaluated the association with the remaining datasets. The second strategy was a standard 10-fold cross-validation applied to each single dataset. The two evaluations yielded the same results (Table 5 and Table S5). Further, we compared the strength of association of the supermarker peak set for gammaretroviral datasets to the performance of the Random Forest machine learning algorithm [94]. The two methods obtained superimposable results (Table S6, see Methods for details).

**Table 5:** Association of supermarker with gammaretroviruses.

		Matched^a^			Unmatched	
Retrovirus	F_0.5_ score	p-value	wi2kb(%)	F_0.5_ score	p-value	wi2kb(%)
MLV HeLa [43]	0.87	3E-285	75	0.61	1E-57	32
MLV HeLa [31]	0.85	<1E-350	70	0.60	1E-88	29
MLV CD4+T [71]	0.84	2E-113	71	0.67	1E-42	39
HIVmINmGAG [43]	0.86	4E-264	70	0.56	1E-24	27
XMRV [76]	0.83	1E-190	66	0.70	1E-85	41
PERV [77]	0.83	<1E-350	66	0.75	<1E-350	51

a Matched means that the supermarker was calculated using proviruses cloned from the same cell type as the ChIPSeq dataset. In the case of XMRV and PERV, proviruses were cloned from a cell type that is similar to the ChIPSeq dataset, according to the transcriptional profile (see text and Figure S2).

**Figure 9:**
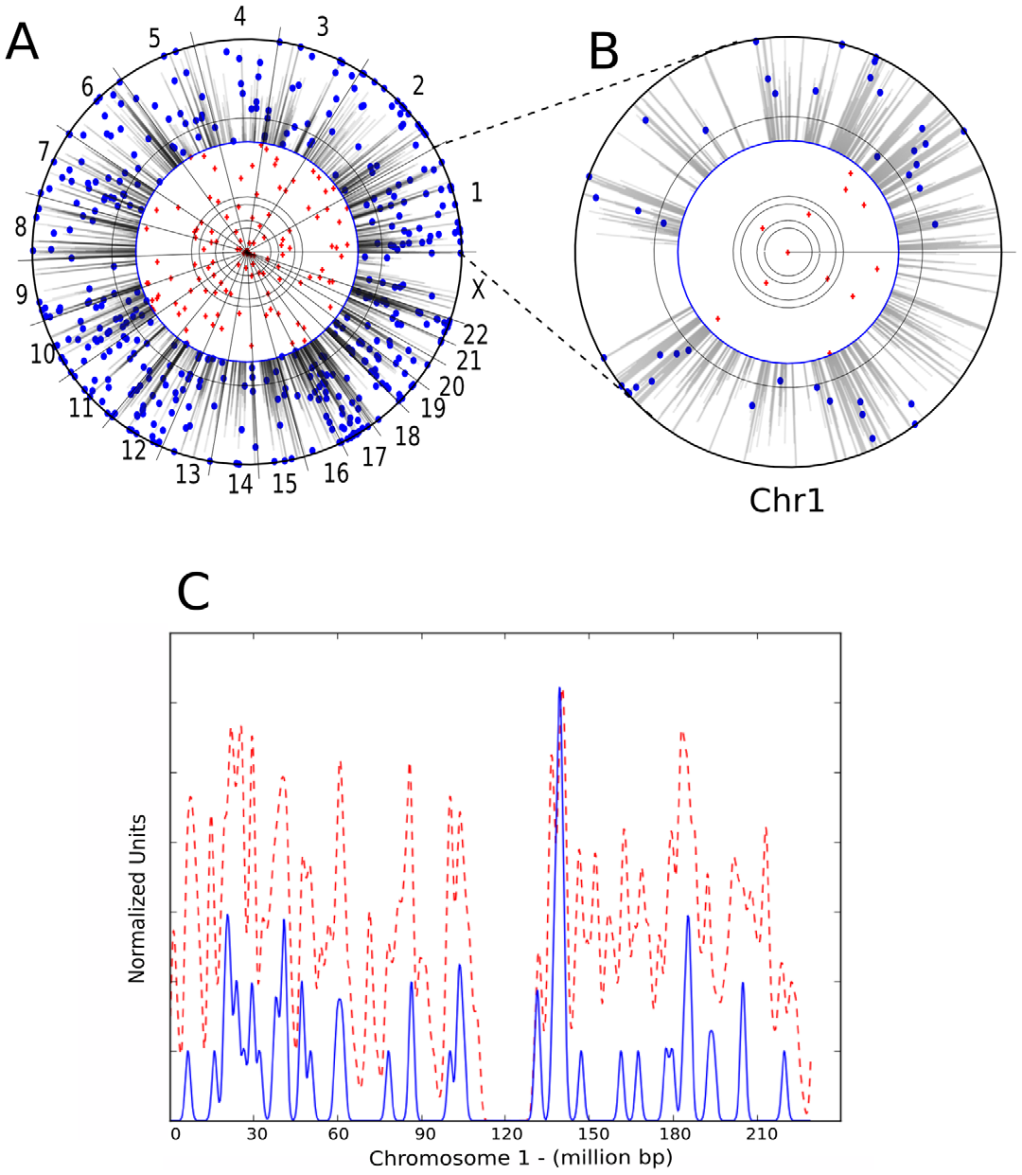
Visualization of association between retroviral integration sites and the chromosomal supermarker. (**A**) Chromosome projection mandala showing MLV proviruses from HeLa cells plotted as in Figure 1 and 2 with supermarker density (gray shading) from the 2 kB circle to the contour of the circle. (**B**) Chromosome projection mandala for chromosome 1 in isolation. (**C**) Plot showing density of supermarker (red dashed line) vs MLV proviruses (solid blue line) in HeLa cells, calculated over a 10 kB sliding window on chromosome 1. Pearson correlation is 0.81 for chromosome 1 and 0.75 for the whole genome.

With respect to MLV integration in HeLa cells, H3K4me1, H3K4me3, H3K9ac and STAT1 were the markers with the best F scores (>0.80)(Table S1 and S2). Examination of the ChIPSeq peaks derived from all combinations of these five candidates revealed that the best supermarker was generated by combining H3K4me3, H3K4me1, and H3K9ac (75% wi2kb; p<10^−284^; F score 0.87) (Figure 9 and Table 5). Figure 9A shows the distribution of supermarker density and MLV integration sites across the human genome, with an expansion of chromosome 1 to help visualize detail in Figure 9B. The Pearson correlation for the supermarker density and MLV integration site density across the whole genome was 0.75 (p = 0, with both functions averaged over a non-overlapping 10 kB window). Figure 9C shows the correlation for chromosome 1 in isolation. As with the single marker H3K4me3, the supermarker yields a maximal F score using a window size of 2 kB (Figure 4).

Inclusion of STAT1 in the HeLa supermarker increased the number of false positives over the number of true positives and thus decreased the composite F score. This suggests that any information carried by STAT1 is contained within the other markers.

Among the ChIPSeq data in CD4^+^ T cells, the best individual markers associated with MLV were H3K4m1, H3K4m2, H3K4m3, H3K9ac, H2BK120ac, H2BK5ac, H3K18ac, H3K27ac, and H2AZ (all >0.80, Table S1 and S2). The best supermarker for MLV on CD4^+^ T cells was composed of H3K4m1, H3K4m2, H3K4m3, and H3K9ac (71% wi2kb; p<10^−122^; F score 0.84).

### The F score detects differences between cell types

The F scores reported here (Tables 3 and 4) were calculated using ChIPSeq and provirus datasets that were matched for cell type. In a previous report, when AUC(ROC) was used to evaluate epigenetic marks mapped in T cells, the correlation with proviruses cloned from T cells was no greater than the correlation with proviruses cloned from other target cell types such as the human embryonic kidney cell line HEK 293 or the fibrosarcoma cell line HT1080 [90]. Differences due to experimental error were in fact greater than differences due to cell type [90].

**Figure 10:**
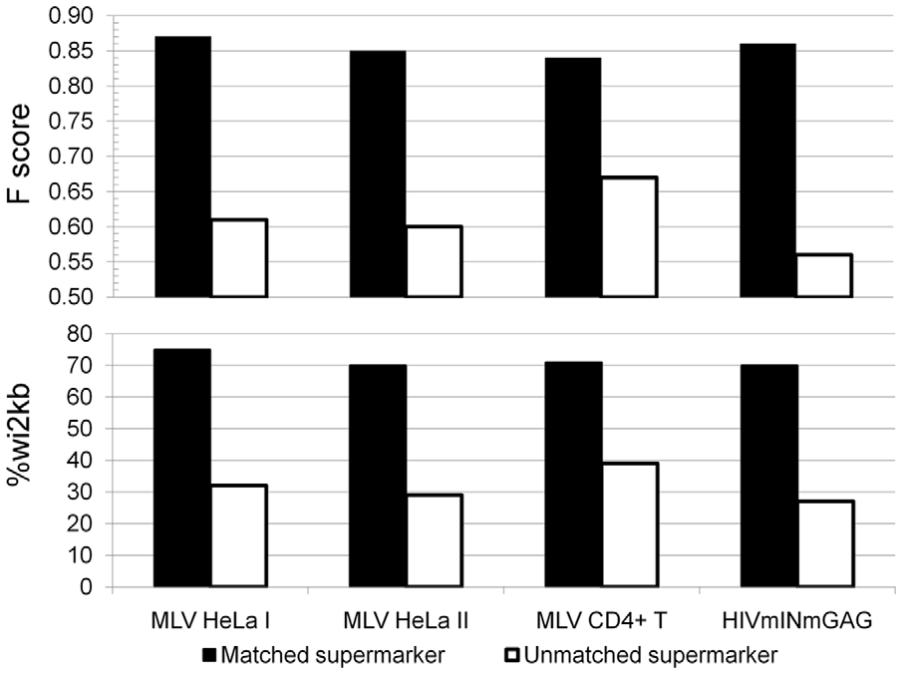
Influence of dataset matching on the F score. Histograms of the F score (upper panel) and the percentage of associated proviruses wi2kb of the supermarkers (lower panel) with respect to MLV proviruses, either from Lewinski et al (MLV HeLa I) or Wu et al (MLV HeLa II), and the HIVmINmGAG chimera, as indicated. Supermarkers were generated with ChiPSeq data from HeLa cells or from CD4+ T cells and compared with MLV proviruses from either HeLa cells or CD4+T cells. “Matched” means that the provirus and the supermarker are from the same cell type.

To determine if the F score has the ability to discriminate between cell types, MLV provirus data sets from HeLa and CD4^+^ T cells were compared with the supermarker for each of these cell types, in all combinations. As mentioned above, when an MLV provirus dataset obtained from infection of HeLa cells [43] was compared with the supermarker from HeLa cell ChIPSeq data, very strong association was observed (75% wi2kB; p<10^−284^; F score 0.87) (Table 5 and Figure 10). When the same provirus dataset was compared with the supermarker derived from CD4^+^ T cell ChIPSeq data the strength of the association was much decreased (32% wi2kB; p<10^−57^; F score 0.61) (Table 5 and Figure 10). The same pattern was seen for the chimera HIVmINmGag, for which association with the supermarker in HeLa cells (70% wi2kB; p<10^−263^; F score 0.86)(Table 5 and Figure 10) was much greater than association with the supermarker in CD4+ T cells (27% wi2kB; p<10^−24^; F score 0.56) (Table 5 and Figure 10). The opposite pattern was also seen in that MLV proviruses cloned from CD4+ T cells [71] were strongly associated with the supermarker derived in these cells (71% wi2kB; p<10^−112^; F score 0.84) (Table 5 and Figure 10), and less well associated with the supermarker from HeLa cells (39% wi2kB; p<10^−42^; F score 0.67) (Table 5 and Figure 10).

A similar analysis was attempted with provirus datasets for the gammaretroviruses XMRV and PERV (Table 5). The XMRV provirus data was obtained in the human prostate cancer cell line DU145 [76] and ChiPSeq datasets are not available for these cells. Despite the mismatched cell lines, when the XMRV dataset from DU145 cells was compared with the epigenetic markers mapped in HeLa cells strong correlation was observed with the supermarker (66% wi2kB; p<10^−190^; F score 0.83). When the supermarker was derived from CD4^+^ T cell data, the association with XMRV was much less significant (41% wi2kB; p<10^−85^; F score 0.70). Similarly, the PERV provirus dataset cloned from HEK 293 cells was better associated with the supermarker from HeLa cells (66% wi2kB; p<10^−350^; F score 0.83) than from CD4+ T cells (51% wi2kB; p<10^−350^; F score 0.75).

To understand why some mismatched cell comparisons gave higher F scores than others, CD4+ T cells, HeLa, DU145, Jurkat, HEK 293, and CD34+ hematopoietic stem cells were clustered based on global gene expression profiles (http://www.ncbi.nlm.nih.gov/geo). The resulting dendrogram (Figure S2) demonstrated that the cells clustered into two groups, one consisting of HeLa, DU146, and HEK 293 cells, and the other CD4+ T cells, Jurkat cells, and CD34+ cells. Based on expression profiles DU145 cells are more similar to HeLa cells than to CD4+ T cells, offering an explanation for the higher F score when XMRV was compared with HeLa.

### Use of the supermarker to predict the likelihood of integration at specific loci within specific cell types

As a first step towards examining the utility of the supermarker in the context of published clinical or experimental data, supermarker density was examined in proto-oncogenes that have been activated by retroviral insertion. 20 SCID-X1 patients were successfully treated with autologous bone marrow CD34+ hematopoietic stem cells transduced ex-vivo with an MLV vector expressing the therapeutic gene *IL2RG*. 5 of these patients developed T cell leukemia and 4 possessed insertional mutations from the MLV vector at *LMO2* [24]–[28], a T cell oncogene [95]. The fifth patient had a provirus near *CCND2*, another lymphoid oncogene [96] that encodes cyclin D2.

**Figure 11:**
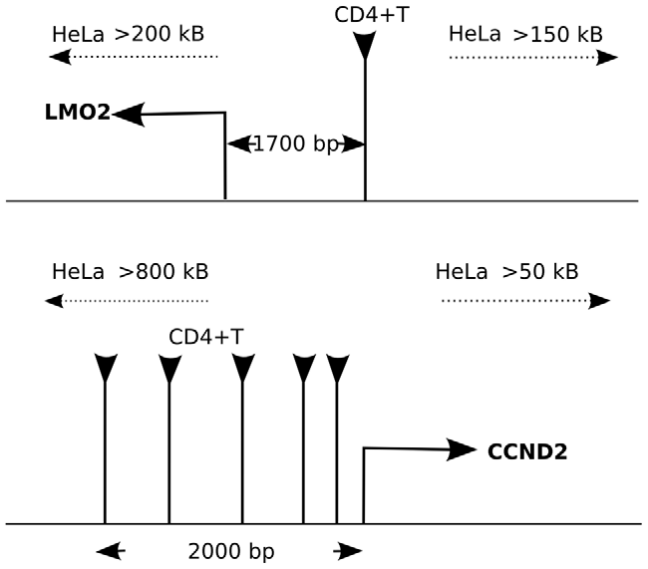
Cell type-dependence of supermarker density near the promoters of protooncogenes. (**A**) Schematic diagram of the region on human chromosome 11 flanking the promoter of the protooncogene *LMO2*. In CD4+ T cells, a very prominent supermarker peak is found wi2kB of the TSS. According to supermarker density, the probability of MLV integration in this region is 1 in 10^5^. In HeLa cells, the nearest supermarker is found more >150 kB upstream and the probability of MLV integration is 1 in 10^7^. (**B**) Schematic diagram of the region on human chromosome 12 flanking the promoter of the protooncogene *CCND2*. In CD4^+^ T cells, a dense cluster of supermarker peaks is found wi2kB of the TSS, and the probability of MLV integration is 1 in 10^4^. In HeLa cells, the nearest supermarker is found >50 kB downstream and the probability of MLV integration is 1 in 10^7^.

When ChIPSeq datasets from HeLa cells were used to generate the supermarker, no high probability sites were identified near the promoters of *LMO2* or *CCND2* (Figure 11). For LMO2 the nearest sites in HeLa cells were >150 kbp upstream and >200 kbp downstream of the TSS. For CCND2 the nearest sites in HeLa were >800 kbp upstream and >50 kbp downstream of the TSS.

Sufficient ChIPSeq datasets to generate a supermarker were not available for CD34^+^ hematopoietic stem cells. Given the relative similarity of the transcription profile (Figure S2) we used the supermarker data generated from CD4^+^ T cells. The F score when crossing from CD34+ cells to CD4+ cells decreases from 0.85 to 0.78 (57% wi2kb, p<10^−102^), but is much better than when using HeLa cell data (38% wi2kB; p<10^−48^ ; F score 0.66).

With respect to the LMO2 TSS a very prominent supermarker peak was observed at −1730 bp (Figure 11A). Based on the probability of the supermarker we estimate that 1 out of 10^5^ MLV proviruses would target this gene in CD34+ cells or CD4+ T cells, as compared to a much less frequent 1 out of 10^7^ MLV proviruses in HeLa cells. Nearly identical probabilities were calculated based on experiments in which MLV proviruses were cloned from T cell lines and HeLa cells [97]. These authors observed a hotspot for MLV integration located between −1740 to −3000 of the *LMO2* promoter within CD4+ T cells but not within HeLa. Though experimental data for calculating the probability of integration into CCND2 is not available, it is interesting that multiple, high-probability supermarkers are located wi2kB of the promoter (Figure 11B).

## Discussion

Here we attempted to identify epigenetic markers predictive of retroviral integration site selection. To this end, the growing body of ChIP-Seq and retroviral integration datasets was exploited. Borrowing from the field of information retrieval, we derived a measure, the F score, that allowed us to identify and rank candidate markers for association with proviruses. Covalent modification of histone H3, most prominently H3K4me1, H3K4me3, and H3K9ac, as well as binding sites for the transcription factor STAT1, were tightly linked to proviruses from MLV, XMRV, and PERV. The F score also permitted us to combine factors to generate a supermarker that predicted 75% of integration sites with precision and with specificity for integration site preference within a given cell type. The ChIPSeq datamining approach used here identified markers for gammaretroviral integration site selection that are superior to any markers previously reported.

### Advantages of the F score

Prior to this study, the best predictor for retroviral integration site selection was the association of TSS/CpG with gammaretroviruses such as MLV [31], [43], [71]. Given a window of 2 kB, TSS/CpG predicts 21 to 27% of MLV integration sites. But even this modest prediction comes with the cost of a high background rate (low precision) and consequently a borderline F score (0.51 under the best conditions). In contrast, H3K4me3 predicts 63 to 68% of MLV integration sites with high precision (F score 0.84). H3K4me1 predicts 90% of MLV integration sites but, in isolation, this marker has a higher background rate (F score 0.78) due to the larger size of the H3K4me1 ChIPSeq dataset (300,000 binding sites for H3K4me1 versus 70,000 for H3K4me3).

Previous studies have reported the same histone modifications as markers associated with integration sites [81], [90]. The Precision-Recall methods used here have been shown to be better suited than ROC when negative results far exceed positive ones [82]. Precision-Recall methods have been shown to perform better than ROC in a number of other areas in biology, including the prediction of functional residues within proteins [98] or predicting the function of genes [99]. In our case, the resolution offered by the Precision-Recall-based F score allowed us to rank markers according to statistical significance (Text S1). Then, by ranking markers with respect to their F score, we were able to combine them to generate a supermarker which predicts 75% of MLV integration sites wi2kB with very high precision (F score 0.87). It will certainly be important to find an explanation for the remaining 25% of integration sites not accounted for by the markers identified here.

### Significance of the supermarker

The supermarker was used here to predict the probability of gammaretroviral integration into a specific locus, in a cell-type specific manner (Figure 11). Our *in silico* probability estimates for integration near a particular proto-oncogene, LMO2, were nearly identical to the probabilities calculated from experimental data [97], and even concurred with respect to the cell-type specificity of the experimentally determined probability. Additional experimental confirmation of supermarker predictions is called for but the case of LMO2 suggests that the supermarker is indeed the first powerfully predictive tool for retroviral integration site selection. A supermarker generated from cell-type-specific ChIPSeq data for a handful of markers has the potential to transform how decisions are made concerning clinical gene-therapy trials.

The calculations here were based on distinct datasets from multiple sources (Tables 1 and 2). It is possible that by generating matched datasets, i.e., integration datasets and ChiPSeq datasets from identical cells and by the same laboratory, or by combining ChIPSeq data for new factors in new combinations, the ability of the supermarker to predict integration sites will be improved even further. On the other hand, STAT1, a powerful marker in isolation, increased the false positive rate and decreased the F score. In addition to the ChIPSeq datasets in Table 1, we checked if the F score was improved by examining other previously reported features, including GC content, AT content, putative consensus sequences for integration or transcription factors [80], [100]. When a window of 2kB was considered, these features failed to yield a significant F score (all were ≤0.5) for all of the retroviral provirus datasets, and these factors considerably lessened the F score when combined with the highly associated markers (Table S7).

### Mechanistic implications

The strength of the associations with H3K4me3, H3K4me1, and H3K9ac indicates that gammaretroviral integration is not a quasi-random process, but rather, a deterministic process that follows the epigenetic histone code. Though some of these histone modifications are linked to transcriptionally active promoters [64], [87]–[89], the link to transcription per se seems not to be relevant since 60 to 70% of supermarker loci are not associated with TSS/CpG. Consistent with this point, our supermarker is highly associated with the LMO2 promoter in CD4+ T cells, but not in HeLa cells, and these cell-type-specific differences in marker binding do not correlate with differential LMO2 expression in these cells [97]. The 2 kB window maxima for the F score of the supermarker is intriguing and suggests that it is a physical property of chromatin that is favored for integration by gammaretroviruses, perhaps linked to the position of the supermarker relative to nucleosomes or bent DNA [34], [36]–[38].

The factors constituting the supermarker, along with the other histone modifications listed in Tables S1 and S2 that are also associated with MLV integration, suggest a mechanistic link between gammaretroviral integration and chromatin-associated complexes with H3K4 methyltransferase and histone acetyltransferase activity. H3K4 methylation is clearly linked with histone acetylation, in that promoters which are methylated are much more likely to become acetylated [65] and knockdown of WDR5, a factor required for H3K4 methylation [101] leads to altered histone acetylation [65], [102]. Methylation may recruit chromatin remodeling complexes [103], [104], the methylated histone may be bound by the acetylases [105], or acetylases may be components of the methylase complex itself [101]. CBP/p300 is associated with H3K4 methyltranferase activity in vivo [106], [107]. ChIPSeq data on acetyltransferases shows a weak but significant association between CBP and MLV integrations in CD4+ T cells (F score 0.68, Table S4). Interestingly, combination of CBP and p300 leads to an aggregated F score of 0.75. Thus, any of these chromatin associated factors, methylated histones, methylases, chromatin remodeling complexes or acetylases are candidates for gammaretroviral IN-binding factors. Interestingly, HIV-1 IN associates with, and is acetylated by, p300 [108] but the p300 ChIPSeq binding profile was not associated with the HIV-1 proviral datasets (F score 0.34).

### Gammaretrovirus association with STAT1

Though very strong association was observed when any of the gammaretroviruses were compared with STAT1 binding sites, adding this transcription factor to the supermarker did not improve the F score. This is perhaps because any retroviral targeting information derived from STAT1 binding sites is already present in the modified histone H3. 90 to 95% of the STAT1 binding sites are in fact within 2 kB of the nearest H3K4me1 site. Our attempts to detect STAT1 binding to MLV IN, or to see effects of STAT1 disruption on MLV infectivity were unsuccessful. Taken together it seems likely that STAT1 itself is not mechanistically involved in gammaretrovirus integration. More likely, STAT1 homes to chromosomal regions that are also preferred targets for integration by these viruses. STAT1 has a complex relationship with the histone acetylase CBP/p300. Acetylation of histones is required for STAT1-mediated transcription [89], [109] but STAT1 itself binds CBP/p300 [110] and is also acetylated and this contributes to its inactivation [111].

### HIV integration site selection

The best single marker for HIV-1 in HeLa cells, H3K4me1, predicted 48% of proviruses wi2kB but with only moderate precision (F score 0.60). Using the F score we were able to detect a stronger association of HIV-1 with H3K4me1 in CD4+ T cells (57% wi2kB, p<10^−71^, F score 0.73) but combining markers in an attempt to generate a supermarker failed to improve the F score. The associations that were observed may be related to HIV-1's propensity to integrate along the length of transcriptionally active genes [81], [90]. Association with histone modifications at active promoters may be detected given short enough gene-length, or a wide-enough window around the provirus (Figure 5). Either way, we were unable to identify a marker capable of predicting HIV-1 integration site selection wi2kB. Perhaps the HIV-1 IN-interacting protein PSIP1/LEDGF/p75 [53]–[55] would be such a factor. Though binding sites have been reported for LEDGF [112], this dataset is limited to 1% of the human genome and cannot be used for a genome-wide association study. LEDGF influences HIV-1 integration site selection in that its disruption causes a shift away from transcriptional units and towards CpG-rich sequences [56], [58], [59]. Nonetheless, these are relatively general effects and LEDGF binding sites may fail to give resolution down to a window of 2 kB. It appears that integration site selection by HIV-1 is mechanistically quite different than for the gammaretroviruses.

## Methods

### Retrovirus integration site datasets and generation of controls

The analysis of integration sites was based on the published integration datasets in Table 2. In the analysis performed here, to control for possible bias introduced during the cloning of the integration sites, 10 control sites in the human genome were generated for each integration site, as previously described [42], [43], [78], [80]. These control, *in silico*-generated sites were used to calculate the significance and the F score (see below).

### CpG island and transcription start sites

These genomic features were obtained from Annotated Genome version hg18 for human (http://genome.ucsc.edu/). CpG island and transcription start sites were combined into single datasets for determining association with retrovirus integration sites.

### ChIPSeq datasets

ChIPSeq peaks were derived from published ChIPSeq datasets (Table 1) with a robust and fast algorithm, F-Seq [113] running with default parameters and standard Poisson statistics. We recalculated the peaks even when the peak set was already available to confirm the reproducibility of published procedures.

### Statistical analysis

Two-sided Fisher exact test (or χ^2^ approximation when appropriate) was used to evaluate statistical significance. All p-values were Bonferroni corrected for multiple testing. p-values<0.01 were considered significant.

To measure marker performance with respect to a given retroviral integration dataset, we used the 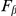-score (van Rijsbergen 1979). It is defined as the β-weighted harmonic mean of Precision 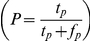 and Recall
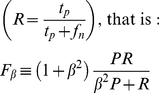
(1)where *tp* is the number of actual integration sites within 2 kB from a specified factor; *tn* is the number of control datapoints (generated *in silico* as described above) >2 kB from a specified factor; *fp* is the number of control datapoints within 2 kB from a specified factor and *fn* is the number of actual integration sites >2 kB from a specified factor. We set β = 0.5 to give more weight to Precision than to Recall. This balances type I and type II errors by adjusting for the high rate of False Positives (*fp*) inherent in the examination of large datasets for genome-wide binding sites according to statistical significance (Text S1). Moreover, to overcome the limitation of standard statistical methods we normalized *fp* with respect to the number of actual integration sites.

The normalized 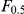-score is finally
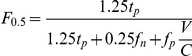
with V and C being, respectively, the number of effective and control integration sites. The resulting F score is almost constant with respect to the size and ratio of experimental and control datasets (Figure 7).

It is worth noting that a null-predictor yielding 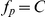 (i.e. a marker composed of all bases in the genome) gives P = 0.5 and R = 1, resulting in an F score
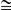
0.5. A marker is considered significant if the F score lies between 0.5 and 1.0.

### Marker ranking and metric comparison

Different metrics can be used to measure the association between proviruses and given markers. We opted to identify the metric among F_0.5_, F_1_, F_2_, Area Under Curve (AUC), Area Under Precision/Recall (AUPR), Odds Ratio (OR), Shannon Mutual Information (SMI), and Difference of Proportions (DOP) that best agrees with statistical significance. The association between markers and proviruses was measured according to each of the above-mentioned metrics. Then the markers were ranked by comparing the measure associated to the i-esim marker with that associated with the j-esim marker and filling in an NXN matrix M for each measure. Formally
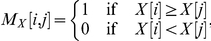
where X is one of the considered metrics. As a reference, a similar matrix was built using the p-value (significance) obtained by Fisher's exact test, defined for the i-esim marker as 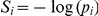. Thus
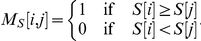
A simple measure of similarity between metric X and reference S was calculated by 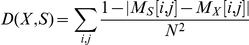 (sum spans over all matrices elements). Observe that 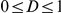.

### Generation of a supermarker

The mass probability functions p(V = i) or p(M = i) are defined as the probability of a provirus V or a marker M to be localized at a given genomic location defined as i≡(chromosome, position). p(V = i) is estimated from the linear combination of mass probability functions for candidate markers, that is
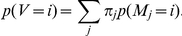
Coefficient 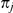 measures the goodness of fit of the marker 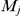 and it seems reasonable to write 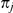 as a function of the related F score.

Indeed the probability of integration P(V) can be written as
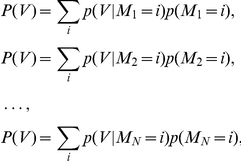
with respect to a set of markers *M_1_,M_2_,…,M_N_*.

Adding these equations we get the mixture model
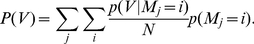
(2)Now, from (1) and 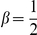 we have
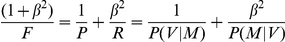
then
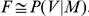
A first order approximation of (2) is then
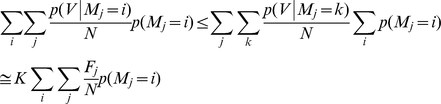
where K is a normalization constant. Eventually we set 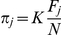 and the resulting new probability mass function is
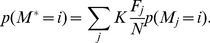
(3)The marker mass density 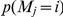 was modeled as the sum of Gaussian functions centered on ChIPSeq peaks, with the variance set as the average size of the peak regions, as determined by the F-seq algorithm [113]. In this way we minimized the potential bias that can arise by summing ChIPSeq densities obtained over different experimental conditions. Briefly, each marker probability density function was written as
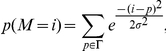
where *Γ* is the peak set of the marker *M*.

This function (3) summarizes the properties of all the markers and can be interpreted as a new ChIPSeq density. Indeed it contains all markers associated and not associated peaks. To reduce the number of false positives we applied a thresholding procedure similar to that used to filter raw ChIPSeq data in a training set of experimental and control integration sites. The peaks of function (3) were ranked with respect to their amplitude and the F score is recalculated on the training set as a function of the number of peaks. We define the supermarker M* as the marker set that yields a maximal F score.

The supermarker density function is finally written as
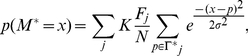
(A)where Γ* is the reduced peak set.

To validate the model, we adopted two strategies. First we calculated the supermarker and the relative reduced peak set on each single proviral dataset and then we evaluated the association with the remaining datasets. The second strategy was a standard 10-fold cross-validation applied to each single dataset.

### Machine learning

To validate the effectiveness of the supermarker peak set, we trained RandomForest [94], a machine learning algorithm, with the same set of markers composing the supermarker. Our datasets are extremely imbalanced and this results in a classifier with an high misclassification error for predicting the minority class (i.e. the experimental dataset) as shown in Table S6. In order to correct for that, RandomForest can be tuned by an additional parameter, *classwt*, that can be used to assign priors to the classes (experimental and control) to minimize the misclassification error and improve the performance. We adopted a 10-fold cross-validation procedure by correcting the priors in the training set. Interestingly, the maximum achievable F score and the number of associated integration sites wi2kb match almost exactly with the F score and wi2kb that we obtained with our supermarker procedure. We consider this as further evidence of the effectiveness of the supermarker.

### Position specific scoring matrix (PWM)

PWM for retroviruses and human transcription factors was borrowed from [80] and from the JASPAR database (jaspar.cgb.ki.se).

### Computation

All computation and graphics were done with ad-hoc Python scripts with the support of the motility library for PWM calculations (*cartwheel.caltech.edu/ motility*), Matplotlib library for graphical and scientific computing (*matplotlib.sourceforge.net*) and the Random Forest implementation on R environment (http://cran.r-project.org/web/packages/randomForest/).

### Graphic representation of data

Chromosome projection mandalas (Figure 1) represent the distribution across of the genome of binding sites for a specific factor or histone modification on the circumference of a circle. Each dot represents a retroviral integration site with the following polar coordinates: angular distance corresponds to genomic location on the indicated chromosome; radial distance from the contour of the circle is the log-scaled distance in nucleotides from the closest marker site. Diagrams have been set to visualize proviruses located between 0 and 1 megabase. Proviruses located more than 1 megabase from the nearest marker accumulate at the center of the mandala.

## Supporting Information

Figure S1 Chromosome projection mandala and F0.5 score calculated within 2 kB for the indicated markers (columns) versus the indicated proviruses (rows). ASLV and HTLV1 proviruses were cloned from HeLa cells, the Foamy virus from CD34+ hematopoietic stem cells (Table 2 and text). H3K4me3 and STAT1 ChIPSeq datasets were from HeLa cells (Table 1). N indicates the number of specific proviral integrations considered for each analysis. The F0.5 score and the percentage of proviruses within 2 kB are presented under each mandala.
(0.35 MB TIF)

Figure S2 Hierarchical clustering applied to the expression profiles of the cell types cited in this study as a measure of similarity. Branch length correlates inversely with similarity, according to the scale bar.
(0.06 MB TIF)

Table S1 Histone acetylation markers and MLV.
(0.04 MB DOC)

Table S2 Histone methylation markers and MLV.
(0.05 MB DOC)

Table S3 HIV-1 versus histone methylation and acetylation.
(0.04 MB DOC)

Table S4 Acetyltransferases, deacetyltransferases, and MLV.
(0.03 MB DOC)

Table S5 Crossvalidation of supermarker association with gammaretroviral proviruses.
(0.03 MB DOC)

Table S6 Comparison of supermarker with random forest algorithm.
(0.03 MB DOC)

Table S7 Association of various genomic features with proviruses, H3K4me3, and H3K4me1.
(0.04 MB DOC)

Text S1 Comparison of Precision/Recall-based methods with Receiver Operating Characteristic Area and other methods, applied to the analysis of provirus datasets.
(0.52 MB PDF)
