# Supplementary figures and images for: Deciphering the Code for Retroviral Integration Target Site Selection
Source: PLoS Comput Biol. 2010 Nov 24;6(11):e1001008. doi: 10.1371/journal.pcbi.1001008 (PMC2991247; doi:10.1371/journal.pcbi.1001008)

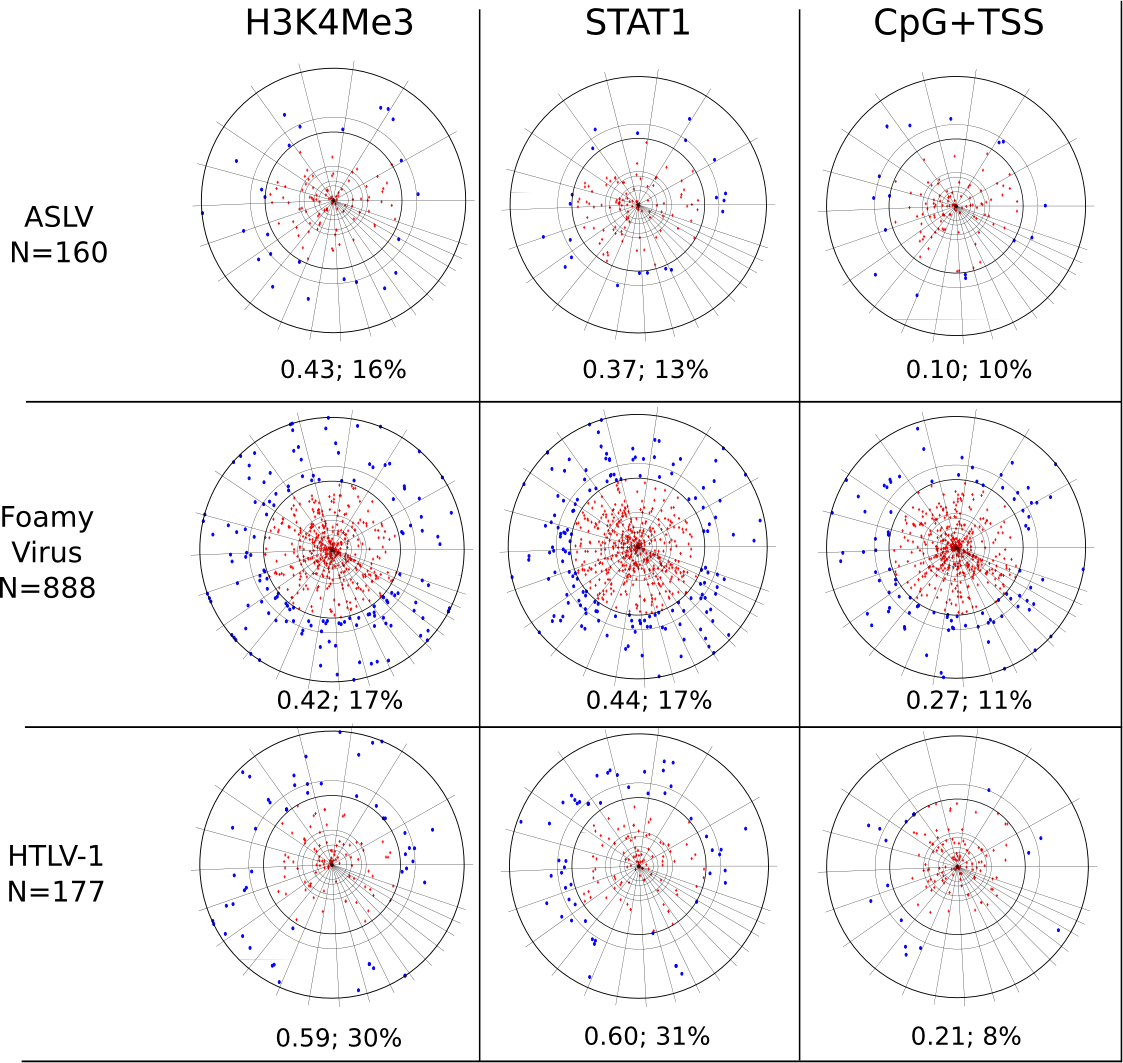

Supplement: Figure S1 — Chromosome projection mandala and F0.5 score calculated within 2 kB for the indicated markers (columns) versus the indicated proviruses (rows). ASLV and HTLV1 proviruses were cloned from HeLa cells, the Foamy virus from CD34+ hematopoietic stem cells (Table 2 and text). H3K4me3 and STAT1 ChIPSeq datasets were from HeLa cells (Table 1). N indicates the number of specific proviral integrations considered for each analysis. The F0.5 score and the percentage of proviruses within 2 kB are presented under each mandala. (0.35 MB TIF) [file pcbi.1001008.s001.tif]

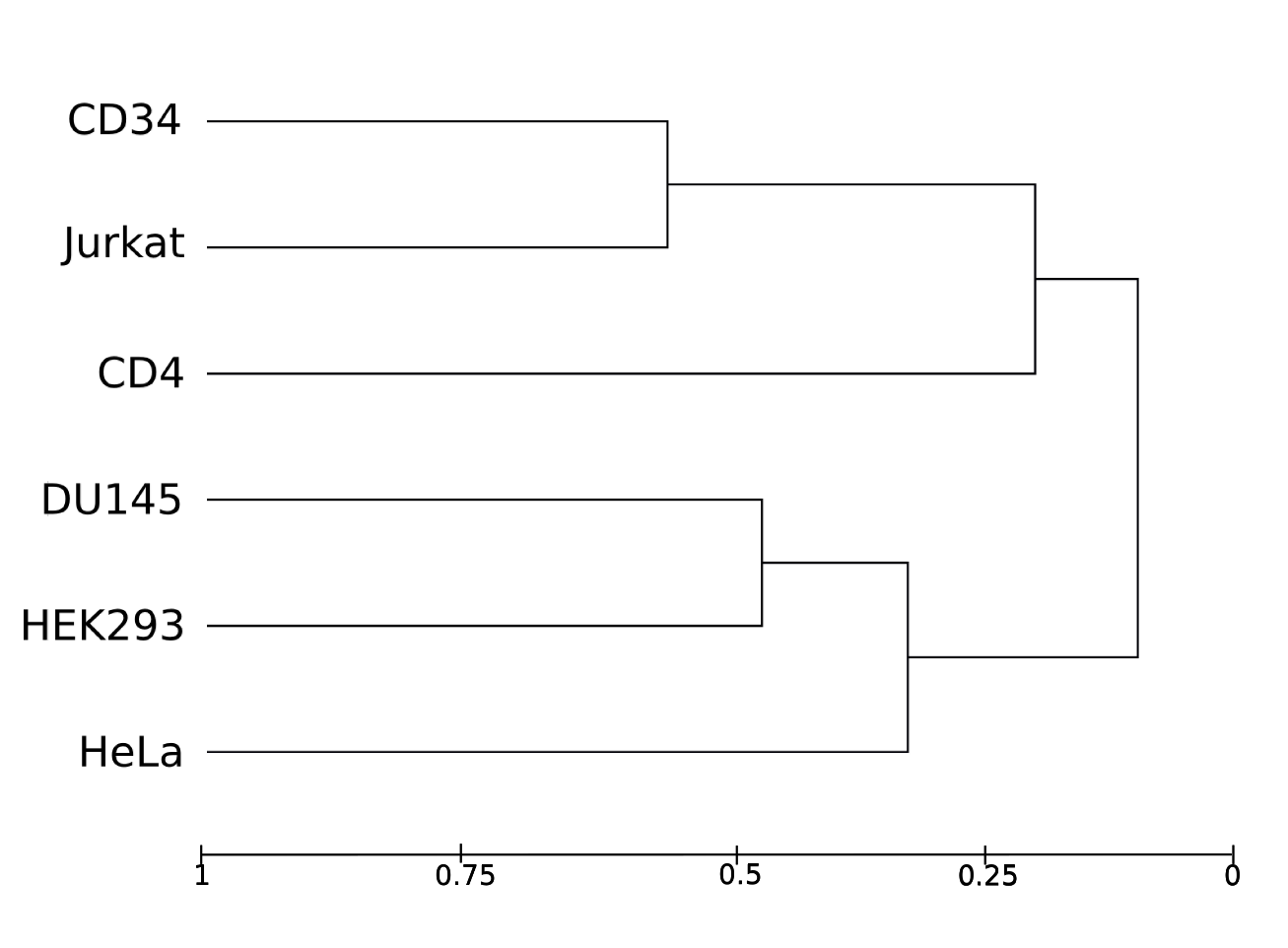

Supplement: Figure S2 — Hierarchical clustering applied to the expression profiles of the cell types cited in this study as a measure of similarity. Branch length correlates inversely with similarity, according to the scale bar. (0.06 MB TIF) [file pcbi.1001008.s002.tif]
