# Supplementary material for: Deciphering the Code for Retroviral Integration Target Site Selection
Source: PLoS Comput Biol. 2010 Nov 24;6(11):e1001008. doi: 10.1371/journal.pcbi.1001008 (PMC2991247; doi:10.1371/journal.pcbi.1001008)
Supplement: Table S1 — Histone acetylation markers and MLV. (0.04 MB DOC) [file pcbi.1001008.s003.doc]

***Table S1. Histone Acetylation [69] and MLV***

| **Modification** | **Cell Type** | **F_0.5_ score** | **^a^Exp vs cont Random %** |
| --- | --- | --- | --- |
| H3K4ac | CD4+ T [71] | 0.78 | 48/3 |
| H3K36ac | CD4+ T [71] | 0.73 | 38/2 |
| H2BK120ac | CD4+ T [71] | 0.83 | 58/4 |
| H3K18ac | CD4+ T [71] | 0.82 | 59/4 |
| H2BK20ac | CD4+ T [71] | 0.79 | 52/4 |
| H2BK5ac | CD4+ T [71] | 0.80 | 55/4 |
| H2BK12ac | CD4+ T [71] | 0.75 | 44/3 |
| H4K8ac | CD4+ T [71] | 0.72 | 39/3 |
| H4K91ac | CD4+ T [71] | 0.79 | 51/4 |
| H4K16ac | CD4+ T [71] | 0.35 | 12/2 |
| H3K27ac | CD4+ T [71] | 0.84 | 60/4 |
| H2AK5ac | CD4+ T [71] | 0.37 | 11/1 |
| H3K16ac | HeLa [43] | 0.73 | 42/6 |
| H3K9ac | CD4+ T [71] | 0.80 | 56/6 |
| H3K9ac | HeLa [43] | 0.82 | 61/7 |

^a^% of experimental proviruses wi2kB versus the % randomized control sites wi2kB
