# Supplementary material for: Deciphering the Code for Retroviral Integration Target Site Selection
Source: PLoS Comput Biol. 2010 Nov 24;6(11):e1001008. doi: 10.1371/journal.pcbi.1001008 (PMC2991247; doi:10.1371/journal.pcbi.1001008)
Supplement: Table S2 — Histone methylation markers and MLV. (0.05 MB DOC) [file pcbi.1001008.s004.doc]

**Table S2. Histone methylation and MLV**

| **Modification** | **Cell Type** | **Virus** | **F_0.5_ score score** | **^a^Exp vs cont** |
| --- | --- | --- | --- | --- |
| H3K4me1 [63] | HeLa | MLV [43] | 0.80 | 90/26 |
| H3K4me3 [63] | HeLa | MLV [43] | 0.84 | 68/10 |
| H3K4me1 [63] | HeLa | MLV [31] | 0.78 | 88/25 |
| H3K4me3 [63] | HeLa | MLV [31] | 0.83 | 63/9 |
| H2AZ [64] | CD4+ T | MLV [71] | 0.82 | 62/7 |
| H2BK5me1 [64] | CD4+ T | MLV [71] | 0.69 | 55/12 |
| H3K27me1 [64] | CD4+ T | MLV [71] | 0.38 | 26/5 |
| H3K36me1 [64] | CD4+ T | MLV [71] | 0.37 | 26/5 |
| H3K36me3 [64] | CD4+ T | MLV [71] | 0.26 | 24/10 |
| H3K79me1 [64] | CD4+ T | MLV [71] | 0.22 | 21/04 |
| H3K79me2 [64] | CD4+ T | MLV [71] | 0.10 | 4/1 |
| H3K79me3 [64] | CD4+ T | MLV [71] | 0.32 | 19/5 |
| H3K9me3 [64] | CD4+ T | MLV [71] | 0.03 | 1/1 |
| H3K9me1 [64] | CD4+ T | MLV[71] | 0.81 | 75/12 |
| H3R2me1 [64] | CD4+ T | MLV [71] | 0.53 | 31/8 |
| H3R2me2 [64] | CD4+ T | MLV [71] | 0.31 | 14/3 |
| H3K27me2 [64] | CD4+ T | MLV [71] | 0.24 | 10/3 |
| H3K27me3 [64] | CD4+ T | MLV [71] | 0.09 | 2/2 |
| H3K4me1 [64] | CD4+ T | MLV [71] | 0.80 | 75/13 |
| H3K4me2 [64] | CD4+ T | MLV [71] | 0.84 | 72/10 |
| H3K4me3 [64] | CD4+ T | MLV [71] | 0.85 | 68/8 |
| H3K9me2 [64] | CD4+ T | MLV [71] | 0.14 | 6/1 |
| H4K20me1 [64] | CD4+ T | MLV [71] | 0.63 | 40/10 |
| H4K20me3 [64] | CD4+ T | MLV [71] | 0.13 | 6/2 |
| H4R3me2 [64] | CD4+ T | MLV [71] | 0.11 | 9/3 |

^a^% of experimental proviruses wi2kB versus the % randomized control sites wi2kB
