# Supplementary material for: Deciphering the Code for Retroviral Integration Target Site Selection
Source: PLoS Comput Biol. 2010 Nov 24;6(11):e1001008. doi: 10.1371/journal.pcbi.1001008 (PMC2991247; doi:10.1371/journal.pcbi.1001008)
Supplement: Table S3 — HIV-1 versus histone methylation and acetylation. (0.04 MB DOC) [file pcbi.1001008.s005.doc]

***Table S3. HIV vs. histone methylation and acetylation***

| **Modification** | **Cell Line** | **Virus** | **F_0.5_ score** | **^a^Exp vs cont** |
| --- | --- | --- | --- | --- |
| H2BK5me1 | Jurkat | HIV [79] | 0.61 | 38/13 |
| H2BK5me1 | CD4+ T | HIV [75] | 0.65 | 40/10 |
| H3K36me3 | Jurkat | HIV [79] | 0.62 | 35/10 |
| H3K36me3 | CD4+ T | HIV [75] | 0.67 | 41/10 |
| H4K20me1 | Jurkat | HIV [79] | 0.62 | 35/10 |
| H3K9me1 | Jurkat | HIV [79] | 0.58 | 38/15 |
| H3K4me1 | Jurkat | HIV [79] | 0.59 | 35/13 |
| H3K4me2 | Jurkat | HIV [79] | 0.55 | 29/11 |
| H3K4me1 | CD4+ T | HIV [75] | 0.73 | 56/14 |
| H3K4me2 | CD4+ T | HIV [75] | 0.66 | 42/11 |
| H3K4me3 | CD4+ T | HIV [75] | 0.5 | 23/10 |
| H4K20me1 | CD4+ T | HIV [75] | 0.67 | 44/10 |
| H3K9me1 | Jurkat | HIV [79] | 0.61 | 37/14 |
| H3K9me1 | CD4+ T | HIV [75] | 0.73 | 56/13 |
| H3K27me1 | Jurkat | HIV [79] | 0.60 | 34/11 |
| H3K27me1 | CD4+ T | HIV [75] | 0.67 | 41/10 |
| H3K4me1 | HeLa | HIV [42] | 0.60 | 47/24 |
| H3K4me1u | HeLa | HIV [42] | 0.60 | 45/23 |
| H3K4me1u | HeLa | HIV mIN [43] | 0.50 | 48/22 |
| H3K4me1 | HeLa | HIV mIN [43] | 0.62 | 50/23 |
| H3K4me1u | HeLa | HIV mGAG [43] | 0.50 | 43/24 |

^a^% of experimental proviruses wi2kB versus the % randomized control sites wi2kB
