# Supplementary material for: Deciphering the Code for Retroviral Integration Target Site Selection
Source: PLoS Comput Biol. 2010 Nov 24;6(11):e1001008. doi: 10.1371/journal.pcbi.1001008 (PMC2991247; doi:10.1371/journal.pcbi.1001008)
Supplement: Table S4 — Acetyltransferases, deacetyltransferases, and MLV. (0.03 MB DOC) [file pcbi.1001008.s006.doc]

***Table S4. Acetyltransferases, deacetyltransferases [65], and MLV [71]***

| **Modification** | **Cell Line** | **Virus** | **F_0.5_ score** | **^a^Exp vs cont** |
| --- | --- | --- | --- | --- |
| p300 | CD4+ T | MLV | 0.57 | 17/1 |
| CBP | CD4+ T | MLV | 0.68 | 32/2 |
| MOF | CD4+ T | MLV | 0.63 | 28/3 |
| TIP60 | CD4+ T | MLV | 0.52 | 25/3 |
| P/CAF | CD4+ T | MLV | 0.59 | 30/4 |
| HDAC1 | CD4+ T | MLV | 0.59 | 31/4 |
| HDAC2 | CD4+ T | MLV | 0.23 | 17/4 |
| HDAC3 | CD4+ T | MLV | 0.46 | 21/3 |
| HDAC6 | CD4+ T | MLV | 0.33 | 28/5 |

^a^% of experimental proviruses wi2kB versus the % randomized control sites wi2kB
