# Supplementary material for: Deciphering the Code for Retroviral Integration Target Site Selection
Source: PLoS Comput Biol. 2010 Nov 24;6(11):e1001008. doi: 10.1371/journal.pcbi.1001008 (PMC2991247; doi:10.1371/journal.pcbi.1001008)
Supplement: Table S5 — Crossvalidation of supermarker association with gammaretroviral proviruses. (0.03 MB DOC) [file pcbi.1001008.s007.doc]

**Table S5. Supermarker association with gammaretroviral integration sites measured with 10-fold crossvalidation^a^**

|  | **Supermarker** |  |
| --- | --- | --- |
| **Provirus Dataset** | **F_0.5_ score** | **wi2kb(%)^b^** |
| MLV HeLa [43] | 0.88 | 76 |
| MLV HeLa [31] | 0.85 | 70 |
| MLV CD4+T [71] | 0.84 | 70 |
| HIVmINmGAG[43] | 0.85 | 71 |
| XMRV [76] | 0.83 | 66 |
| PERV [77] | 0.83 | 65 |

^a^each provirus dataset was randomly partitioned into 10 subsets. Each subset was examined for association with the supermarker calculated on the remaining 9 subsets.

^b^Global F_0.5_ score and % of proviruses wi2kb.
