# Supplementary material for: Deciphering the Code for Retroviral Integration Target Site Selection
Source: PLoS Comput Biol. 2010 Nov 24;6(11):e1001008. doi: 10.1371/journal.pcbi.1001008 (PMC2991247; doi:10.1371/journal.pcbi.1001008)
Supplement: Table S6 — Comparison of supermarker with random forest algorithm. (0.03 MB DOC) [file pcbi.1001008.s008.doc]

**Table S6. Supermarker vs. Random Forest^a^**

|  | **Supermarker** |  | **Random Forest** |  | **Random Forest (*classwt*^b^)** |  |
| --- | --- | --- | --- | --- | --- | --- |
| **Retrovirus** | **F_0.5_ score** | **wi2kb(%)** | **F_0.5_ score** | **wi2kb(%)** | **F_0.5_ score** | **wi2kb(%)** |
| MLV HeLa [43] | 0.87 | 75 | 0.80 | 51 | 0.87 | 75 |
| MLV HeLa [31] | 0.85 | 70 | 0.80 | 47 | 0.84 | 69 |
| MLV CD4+T [71] | 0.85 | 71 | 0.78 | 53 | 0.85 | 70 |
| HIVmINmGAG [42] | 0.86 | 70 | 0.76 | 49 | 0.85 | 68 |
| XMRV [76] | 0.83 | 66 | 0.71 | 39 | 0.83 | 67 |
| PERV [77] | 0.83 | 66 | 0.81 | 54 | 0.83 | 66 |

^a^Supermarker and the Random Forest algorithm [91] were trained on the same marker set. Shown is the strength of association as measured by the F_0.5_ score and % of proviruses wi2kb.

^b^this parameter adjusts for imbalanced datasets containing large a excess of true negatives (see methods). A 10-fold crossvalidation strategy was adopted to maximize the F_0.5_ score.
