# Supplementary material for: Deciphering the Code for Retroviral Integration Target Site Selection
Source: PLoS Comput Biol. 2010 Nov 24;6(11):e1001008. doi: 10.1371/journal.pcbi.1001008 (PMC2991247; doi:10.1371/journal.pcbi.1001008)
Supplement: Table S7 — Association of various genomic features with proviruses, H3K4me3, and H3K4me1. (0.04 MB DOC) [file pcbi.1001008.s009.doc]

Table S7. Association of some genomic features with proviral integration sites, H3K4me3 and H3K4me1 ChIPSeq data

|  | **Integration Density^a^ %** | **Control Density %** | **Integration F_0.5_ score** | **p-value (not corrected)^b^** | **H3K4me3 F_0.5_ score** | **H3K4me1 F_0.5_ score** |
| --- | --- | --- | --- | --- | --- | --- |
| MLV motif^c^ [80] | 0.29 | 0.28 | ≤0.50 | >0.1 | 0.51 | ≤0.50 |
| HIV motif [80] | 0.27 | 0.28 | ≤0.50 | >0.1 | ≤0.50 | ≤0.50 |
| MA0056^d^ | 0.68 | 0.80 | 0.51 | 0.08 | 0.52 | ≤0.50 |
| MA0098 | 0.81 | 0.81 | ≤0.50 | >0.1 | ≤0.50 | ≤0.50 |
| MA0081 | 0.82 | 0.83 | ≤0.50 | >0.1 | ≤0.50 | ≤0.50 |
| MA0080 | 0.55 | 0.56 | ≤0.50 | >0.1 | ≤0.50 | ≤0.50 |
| MA0053 | 0.57 | 0.60 | ≤0.50 | >0.1 | ≤0.50 | ≤0.50 |
| MA0020 | 0.75 | 0.77 | ≤0.50 | >0.1 | ≤0.50 | ≤0.50 |
| MA0038 | 0.01 | 0.01 | ≤0.50 | >0.1 | ≤0.50 | ≤0.50 |
| MA0087 | 0.23 | 0.24 | ≤0.50 | >0.1 | ≤0.50 | ≤0.50 |
| MLV AT content^e^ | 54 | 58 | ≤0.50 | 0.03 * | ≤0.50 | ≤0.50 |
| MLV GC content^f^ | 46 | 42 | 0.52 | 0.03 | 0.53 | ≤0.50 |
| HIV AT content | 60 | 58 | 0.51 | 0.09 | ≤0.50 | 0.51 |
| HIV GC content | 40 | 42 | ≤0.50 | 0.1 * | ≤0.50 | ≤0.50 |

^a^Feature densities are calculated wi2kB of MLV or HIV proviral integration sites and H3K4m1 and H3K4me3 ChIPSeq putative sites in HeLa cells.

^b^P-value related to the association of the feature with the proviral integration dataset.

^c^MLV and HIV-1 consensus integration site according to [80].

^d^JASPAR accession codes of Position Weight Matrices of 8 transcription factor binding motifs previously reported to be associated with MLV proviruses in HeLa [100].

^e^% of A and T in genomic DNA sequence wi2kB of proviruses.

^f^% of G and C wi2kB of proviruses.

* indicates a negative association
