# Supplementary material for: Deciphering the Code for Retroviral Integration Target Site Selection
Source: PLoS Comput Biol. 2010 Nov 24;6(11):e1001008. doi: 10.1371/journal.pcbi.1001008 (PMC2991247; doi:10.1371/journal.pcbi.1001008)
Supplement: Text S1 — Comparison of Precision/Recall-based methods with Receiver Operating Characteristic Area and other methods, applied to the analysis of provirus datasets. (0.52 MB PDF) [file pcbi.1001008.s010.pdf]

Supplementary Text S1 for:

Deciphering the code for retroviral integration target site selection

Federico Andrea Santoni[1,2,3], Oliver Hartley[4], and Jeremy Luban[1,5]

Comparison of Precision/Recall-based methods with Receiver Operating Characteristic Area and other methods, applied to the analysis of provirus datasets.

It was the goal of our study to identify markers that are significantly associated with retroviral integration. In the past, several groups have performed this kind of study by using the standard Fisher exact test [31,43,70-77]. Theoretically Fisher p-values are the correct tool to evaluate the association strength of the single marker. Unfortunately p-values are influenced by the experimental and control dataset size and, while reliable in the context of a single dataset, cannot be used as a universal measure (reference 78

and our manuscript Fig. 6 and text). For the purpose of this kind of analysis, the lab of Rick Bushman has also utilized ROC and logistic regression techniques [80,81,90].

Thus our aim was to find a measure with range [0,1], that is invariant with respect to dataset size, but that maintains a strong link with significance. In the manuscript (Fig. 7) we showed that Area Under Curve, the ROC-based measure used by the Bushman Lab [80,81,90], does not satisfy this criteria since, for the reasons reported in references 82,98,99 and in the manuscript [lines 160-170], it is biased with respect to the number of true negatives. This results in a “distortion” with respect to significance.

Indeed, ROC uses the false positive rate fp/(fp+tn) (fp - false positives, tn - true negatives). If the number of true negatives is much higher than the number of false positives “ a large change in the number of false positives can lead to a small change in the false positive rate used in ROC analysis.”[82].

Precision, or positive predictive value, is defined as tp/(tp+fp) (tp – true positives, fp – false positives) so the effect of false positives is directly compared to true positives. Thus, we proposed a standard measure, the $F_{0.5}$ score, based on Precision and Recall (or true positive rate). This measure, which is explained in detail in the methods, is defined independently of PR curves.

We compared ROC and PR curves for all of the markers. In these comparisons, the same set of confusion matrices, calculated with the same cutoff points, were utilized for ROC and PR. As one example (Figure A) we show the ROC and PR curves of two markers, H3K4me1 (blue) and H3K4me3 (dashed red), that are strongly associated with

MLV in HeLa cells [43]. Within the ROC space it is difficult to distinguish by eye which marker is better than the other. According to Area Under Curve (AUC) methods from the Bushman lab [80, 90] H3K4me1 is considered superior to H3K4me3 (and the difference was significant according to the Wald test in the supplement to reference 90). In contrast, in the PR space, it is quite clear that H3K4me3 performs better than H3K4me1. Given the discrepancy between these two methods, what criteria should be used to decide which method is "correct"?

It was our goal to find an index with a strong relationship to significance. The significance -log(p), calculated with the Fisher exact test, of H3K4me3 and H3K4me1 in this specific HeLa proviral dataset is, respectively, 249 and 226. Using this criterion, in this specific comparison, AUPR fits significance better than does AUC.

The comparison of H3K4me3 with H3K4me1 described above is one specific case. In the case of other markers, to see if AUPR fits significance better than AUC, we analyzed one of the MLV integration datasets in HeLa cells [43] (same results are obtained using the other HeLa dataset [31]) and the MLV integration dataset in CD4+ T cells [71] by comparing the strength of association of 9 significant markers (in terms of p-value) from HeLa cells, and 31 significant markers from CD4+ T cell, using different window sizes (2K, 5K, 10K, and 20K basepairs). We calculated ROC and PR curves for all 40 markers, and made all 1600 comparisons using the calculated AUC and AUPR. Then we ranked the markers by comparing the i-esim marker with the j-esim marker and filling in an NXN matrix M for each measure:

$$M_{AUC}[i,j] = \begin{cases} 1 & \text{if} \quad AUC[i] \geq AUC[j] \\ 0 & \text{if} \quad AUC[i] < AUC[j] \end{cases}$$

$$M_{AUPR}[i,j] = \begin{cases} 1 \text{ if } AUPR[i] \geq AUPR[j] \\ 0 \text{ if } AUPR[i] < AUPR[j] \end{cases}$$

In general a similar matrix could be built for a general metric X as:

$$M_X[i,j] = \begin{cases} 1 \text{ if } X[i] \geq X[j] \\ 0 \text{ if } X[i] < X[j] \end{cases}$$

As a reference, a similar matrix was built using the p-value (significance) obtained by Fisher's exact test, defined for the i-esim marker as $S_i = -\log(p_i)$:

$$M_S[i,j] = \begin{cases} 1 \text{ if } \mathrm{S}[i] \geq S[j] \\ 0 \text{ if } S[i] < S[j] \end{cases} .$$

A simple measure of similarity between the ranking obtained by the metric X and that yielded by reference S was calculated as $D(X,S) = \sum_{i,j} \frac{1 - |M_S[i,j] - M_X[i,j]|}{N^2}$ (sum spans over all matrix elements). Observe that $0 \leq D \leq 1$.

Results have been reported in Table 4 and Fig. 8, where red squares indicate that the ranking calculated by the specified metric differs from the rank obtained by significance.

Table 4 shows D(AUC,S) and D(AUPR,S). It is clear that AUPR has a better fit with significance than does AUC, in all the measurements. As discussed below, comparison was also made with other metrics, including Odds Ratio (OR), $F_{0.5}$, F_1, F_2, Shannon Mutual Information (SMI) and and difference of proportions (DOP).

It is worth pointing out that the markers on the axes in Fig. 8 have been ranked by significance. This means that the group of markers that are most highly associated are in the upper-left of the matrix, while the less tightly associated markers are in the lower

right. AUC more commonly diverges from significance when one considers the most highly associated markers (upper left). AUC overestimates association with markers that are more common in the genome due to a higher false positive rate for these markers.

Though AUPR fits our purposes better than does AUC, AUPR suffers from some important limitations. Most of the publicly available proviral datasets have <1000 proviruses and, when the specified window size for analysis is relatively small (generally we use 2kB), there are far too few datapoints available for the construction of PR curves. The problem of too few datapoints cannot be resolved by interpolation because the AUPR is not reliable under these conditions [82]. For this reason, we adopted the F_β score. It considers how many proviruses from a given dataset are within 2 kB of at least one ChIPSeq peak for a particular marker without concern for the number of markers within 2 kB. Given that the F_β score is not subject to the same limitations concerning dataset size as AUPR, the two parameters which are used to derive it, Precision and Recall, can be easily calculated for all markers and all datasets.

When compared to other measures (AUC, AUPR, OR, SMI, and DOP) the F_β score has an advantage which is that it has an additional degree of freedom, beta. Usual values for beta are 0.5, 1, or 2 [84]. A beta value of 0.5 gives more weight to precision; 2 gives more weight to recall. To decide which beta value was best to use, we compared the performance of the F_β score with respect to significance using beta=0.5, 1 or 2. Table 4 reports the percentage of correct ranking of factors with respect to significance for each measure in HeLa and CD4+ T cell datasets. $F_{0.5}$ and SMI are better than AUPR, AUC and the other indexes but SMI has the drawback that it is not invariant with respect to

dataset size. As expected, F_2 and AUC give the poorest performances, reflecting their emphasis of recall (the true positive rate).

As reported in the manuscript (lines 297-302) AUC works better with markers that are not very highly associated with proviruses than for markers that are highly associated (for example, see the lower right corner of the matrices in Fig. 8). This is also confirmed by repeating the ranking test with HIV-1, HTLV, FV, ASLV. As an example, results for HIV are reported in Table A (see below). In these conditions, $F_{0.5}$ keeps good performances while SMI is the worst metric here.

All these results clearly demonstrate that the $F_{0.5}$ score is a robust and suitable measure in this context and performs better than the other measures presented here.

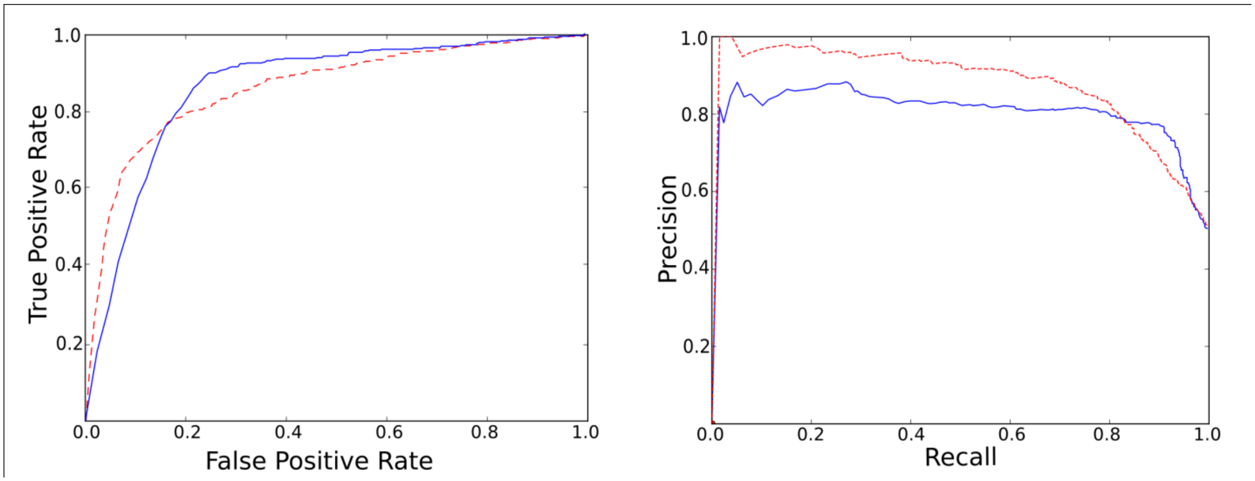

Figure A - Plot of curves generated using ROC (left) or PR (right) for H3K4me1 (blue) and H3K4me3 (dashed red)

Table A. Comparison of different methods for ranking markers[a] of HIV-1 integration in HeLa cells [43]

	AUC	AUPR	$F_{0.5}$	F_1	F_2	OR	SMI	DOP
2K	0.97	0.97	0.97	0.91	0.93	0.97	0.70	0.90
5K	0.97	0.95	0.95	0.93	0.93	0.97	0.83	0.90
10K	0.95	0.97	0.93	0.90	0.85	1.00	0.97	0.85
20k	0.90	0.97	0.90	0.85	0.85	1.00	1.00	0.85

[a]similarity of the ranking of integration markers obtained by each metric with that yielded by Fisher's statistical significance. The formula used to calculate the similarity is in the methods. By this formula, $0 \le D \le 1$, and D = 1 when the ranking perfectly matches that obtained by significance.
